# Supplementary material for: Widespread slowdown in short-term species turnover despite accelerating climate change
Source: Nat Commun. 2026 Feb 3;17:1450. doi: 10.1038/s41467-025-68187-1 (PMC12886849; doi:10.1038/s41467-025-68187-1)
Supplement: Supplementary file 1 — Supplementary Information [file 41467_2025_68187_MOESM1_ESM.pdf]

# Supplementary Information

## for

## Widespread slowdown in short-term species turnover despite accelerating climate change

Emmanuel C. Nwankwo[1], Axel G. Rossberg[1,*]

[1] Centre for Biodiversity and Sustainability, School of Biological and Behavioral Sciences, Queen Mary University of London, Mile End Road, London E1 4NS, UK

[*] Correspond in Author: Axel G. Rossberg, a.rossberg@qmul.ac.uk

This PDF includes:

- Supplementary Figures S1 – S5
- Supplementary Tables S1 – S3
- Supplementary Note 1

## Supplementary Figures and Table

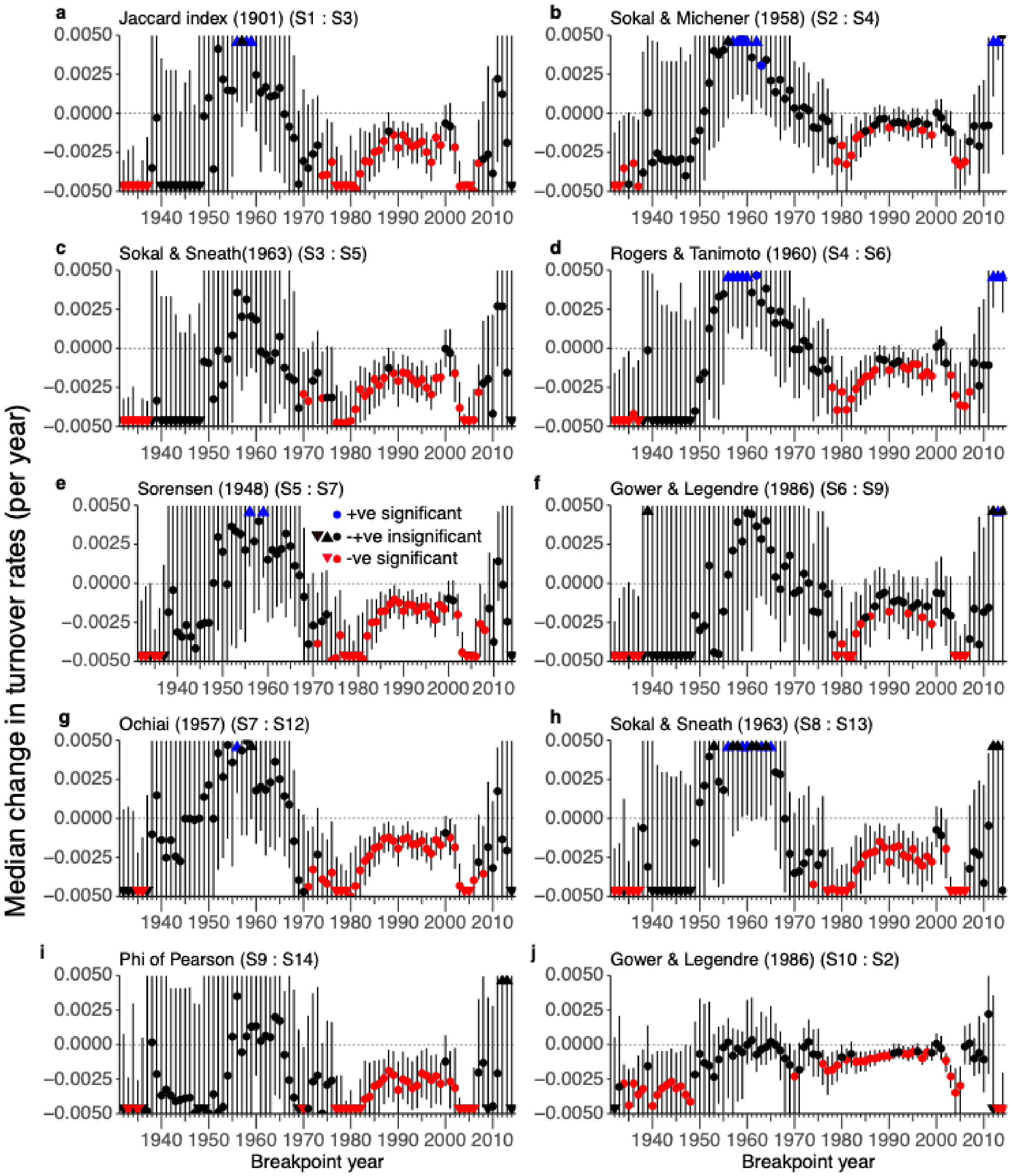


**Fig. S1.** Robustness of our main result to variation in the metrics of community similarity. Each panel shows results for one of the community similarity metrices available in the ade4 R package. Next to the names we include for each metric the pair '(Script ID: Coefficient ID)' based on the numbering by Gower & Legendre (1986). Other than varying similarity metrics, symbols, colouring, and bars indicating 95% confidence intervals are as in Figure 4b of the main text. Variation of metrices produced overall similar patterns in the dependence of change of turnover rate depending on breakpoint year.

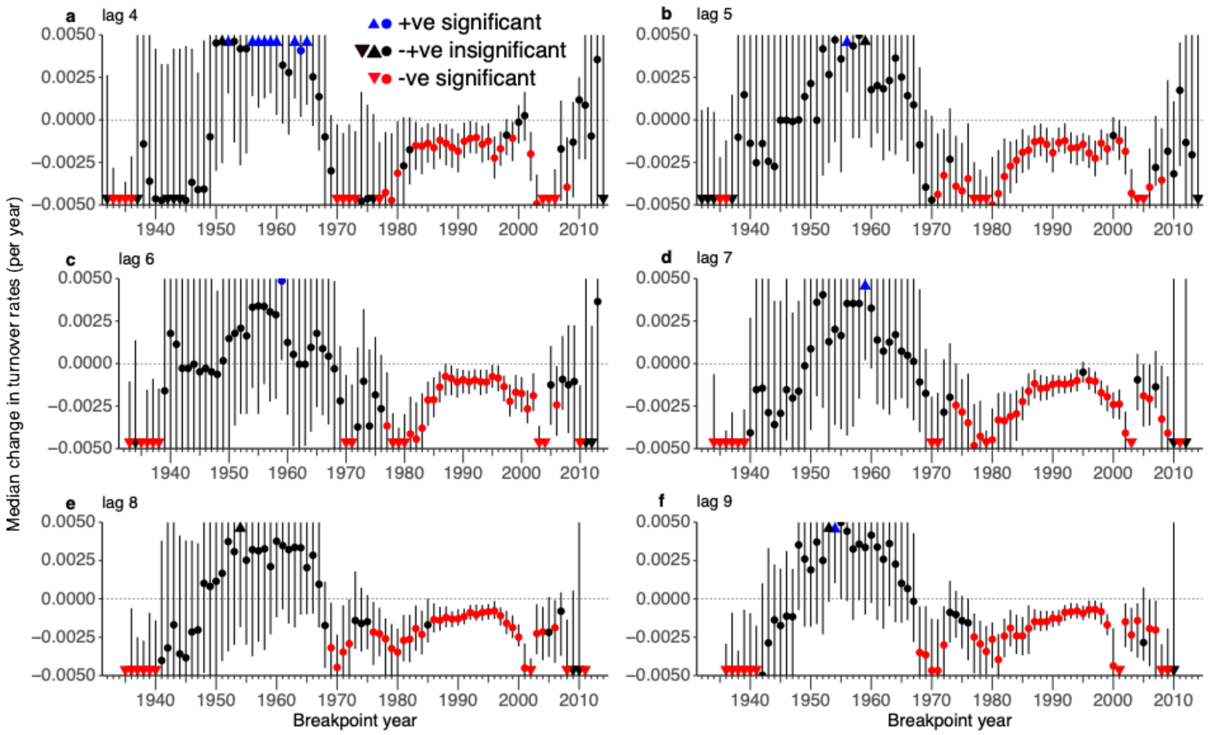


**Fig. S2:** Results of repeating our analysis when increasing the maximum lag included to compute turnover rates from the dependence of community similarity on lag. Each panel corresponds to a different maximum lag as indicated. Symbols as in Figure 4b of the main text.

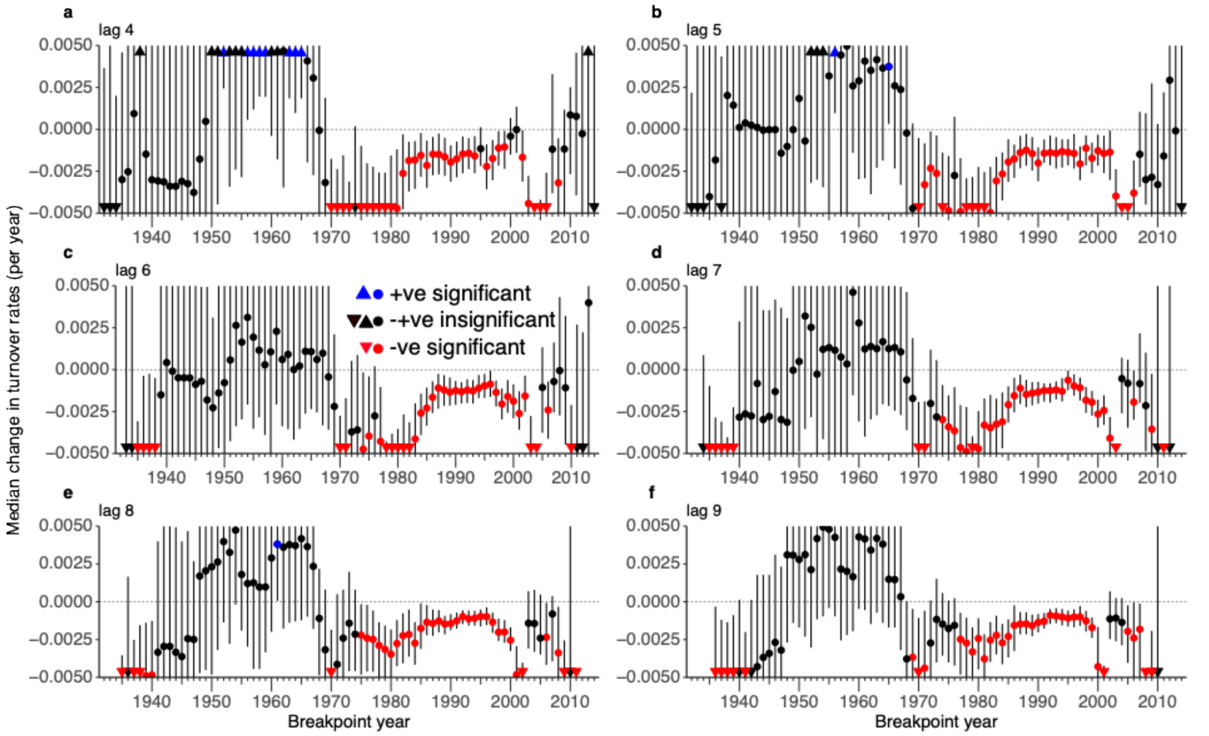


**Fig. S3:** Same as Fig. S2, but after omitting from the published data partitioning protocol a step filtering for `sample completeness', demonstrating that the impact of this step on our results is small. Each panel corresponds to a different maximum lag as indicated. Symbols as in Figure 4b of the main text.

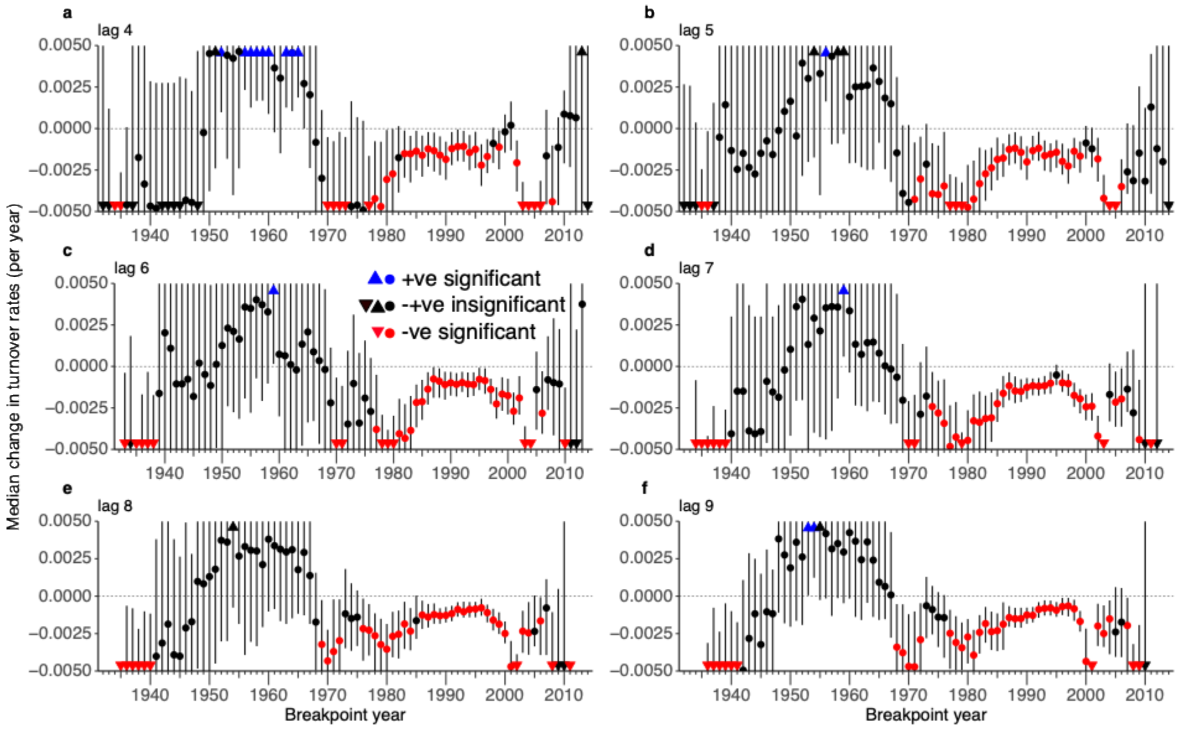


**Fig. S4:** Same as Fig. S2, but after removing all studies for which BioTIME registers a `treatment', demonstrating that the impact of this step on our results is small. Each panel corresponds to a different maximum lag as indicated. Symbols as in Figure 4b of the main text.

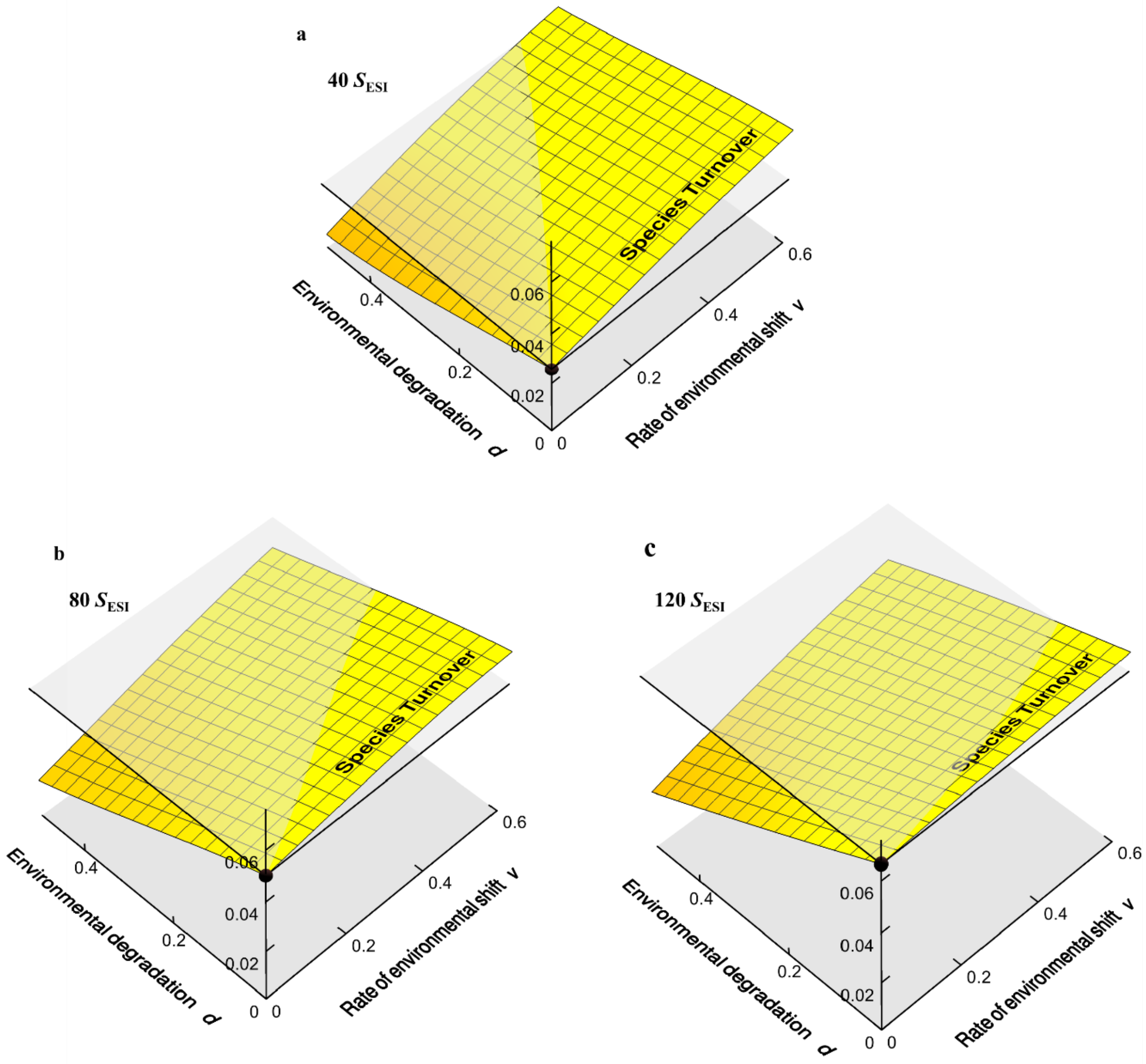


**Fig. S5:** Impact of simulated species pool size on ecological structural stability and species turnover. The three panels correspond to three different pool sizes as indicated (a: 40 $S_{ESI}$, b: 80 $S_{ESI}$, c: 120 $S_{ESI}$, with $S_{ESI}$ = 18.375). Underlying simulations and data analyses are as for Figure 6c of the main text. Larger pool sizes lead overall to faster species turnover, but without any qualitative change in outcomes.

**Table S1.** Median change in community turnover per year across different breakpoint years, with 95% confidence intervals (exact confidence interval of median), sample sizes n, and p-values (two-sided). No correction for multiple testing as these are not independent test. Estimates of median turnover before and since the breakpoint year and percentage of turnover rate decline are shown as well.

| Breakpoint year | median | lower CI | upper CI | *n* | median before breakpoint year | median since breakpoint year | percentage change | p-value |
|---|---|---|---|---|---|---|---|---|
| **1932** | -0.0169 | -0.0325 | 0.0006 | 18 | 0.0221 | 0.0067 | -69.8428 | 0.0963 |
| **1933** | -0.0075 | -0.0247 | 0.0007 | 19 | 0.0123 | 0.0073 | -40.7344 | 0.0636 |
| **1934** | -0.0057 | -0.0136 | 0.0005 | 21 | 0.0132 | 0.0073 | -44.4666 | 0.0784 |
| **1935** | -0.0073 | -0.0134 | -0.0022 | 21 | 0.0158 | 0.0072 | -54.3399 | **0.0015** |
| **1936** | -0.0058 | -0.0123 | -0.0012 | 21 | 0.0150 | 0.0072 | -52.1159 | **0.0266** |
| **1937** | -0.0103 | -0.0201 | 0.0015 | 34 | 0.0157 | 0.0060 | -61.4130 | 0.0576 |
| **1938** | -0.0010 | -0.0115 | 0.0190 | 93 | 0.0176 | 0.0096 | -45.1403 | 1.0000 |
| **1939** | 0.0015 | -0.0089 | 0.0173 | 158 | 0.0059 | 0.0097 | 64.4435 | 0.9366 |
| **1940** | -0.0014 | -0.0095 | 0.0098 | 196 | 0.0115 | 0.0084 | -27.4547 | 0.9431 |
| **1941** | -0.0025 | -0.0073 | 0.0082 | 210 | 0.0111 | 0.0080 | -27.9426 | 0.8361 |
| **1942** | -0.0014 | -0.0095 | 0.0089 | 211 | 0.0115 | 0.0083 | -28.3848 | 0.8905 |
| **1943** | -0.0024 | -0.0088 | 0.0082 | 214 | 0.0114 | 0.0080 | -29.8589 | 0.8376 |
| **1944** | -0.0027 | -0.0088 | 0.0073 | 215 | 0.0115 | 0.0078 | -32.5890 | 0.7851 |
| **1945** | 0.0000 | -0.0095 | 0.0082 | 215 | 0.0115 | 0.0083 | -28.3848 | 1.0000 |
| **1946** | 0.0000 | -0.0093 | 0.0082 | 215 | 0.0107 | 0.0077 | -27.8642 | 1.0000 |
| **1947** | -0.0001 | -0.0126 | 0.0082 | 215 | 0.0104 | 0.0078 | -25.4578 | 0.7851 |
| **1948** | 0.0000 | -0.0086 | 0.0091 | 213 | 0.0097 | 0.0086 | -11.4301 | 1.0000 |
| **1949** | 0.0014 | -0.0050 | 0.0128 | 216 | 0.0067 | 0.0063 | -6.2888 | 0.7338 |
| **1950** | 0.0021 | -0.0064 | 0.0151 | 208 | 0.0055 | 0.0066 | 20.8299 | 0.8353 |
| **1951** | 0.0000 | -0.0065 | 0.0130 | 209 | 0.0048 | 0.0065 | 34.4192 | 1.0000 |
| **1952** | 0.0042 | -0.0033 | 0.0169 | 212 | 0.0039 | 0.0077 | 98.6718 | 0.3029 |
| **1953** | 0.0027 | -0.0025 | 0.0199 | 215 | 0.0083 | 0.0070 | -15.3710 | 0.2195 |
| **1954** | 0.0047 | -0.0013 | 0.0182 | 208 | 0.0066 | 0.0069 | 5.8379 | 0.1876 |
| **1955** | 0.0036 | -0.0031 | 0.0199 | 199 | 0.0077 | 0.0076 | -1.2269 | 0.1561 |
| **1956** | 0.0080 | 0.0016 | 0.0365 | 195 | 0.0072 | 0.0094 | 31.0562 | **0.0098** |
| **1957** | 0.0043 | -0.0011 | 0.0199 | 176 | 0.0089 | 0.0080 | -10.9578 | 0.0592 |
| **1958** | 0.0050 | -0.0011 | 0.0218 | 178 | 0.0082 | 0.0081 | -0.2479 | 0.0606 |
| **1959** | 0.0055 | 0.0000 | 0.0221 | 163 | 0.0082 | 0.0093 | 13.1730 | 0.0598 |
| **1960** | 0.0018 | -0.0016 | 0.0171 | 139 | 0.0062 | 0.0085 | 37.7273 | 0.1745 |
| **1961** | 0.0020 | -0.0008 | 0.0218 | 132 | 0.0047 | 0.0067 | 42.6823 | 0.3384 |
| **1962** | 0.0018 | -0.0024 | 0.0161 | 127 | 0.0055 | 0.0064 | 15.4676 | 0.3750 |
| **1963** | 0.0023 | -0.0018 | 0.0092 | 119 | 0.0059 | 0.0063 | 7.2224 | 0.3594 |
| **1964** | 0.0036 | -0.0021 | 0.0098 | 111 | 0.0050 | 0.0063 | 25.1205 | 0.3426 |
| **1965** | 0.0025 | -0.0012 | 0.0154 | 105 | 0.0059 | 0.0063 | 6.4117 | 0.0785 |
| **1966** | 0.0014 | -0.0024 | 0.0094 | 113 | 0.0075 | 0.0063 | -16.1654 | 0.5727 |
| **1967** | 0.0009 | -0.0032 | 0.0094 | 123 | 0.0076 | 0.0059 | -21.9378 | 0.7185 |
| **1968** | -0.0015 | -0.0053 | 0.0031 | 183 | 0.0111 | 0.0068 | -38.2875 | 0.7676 |
| **1969** | -0.0040 | -0.0121 | 0.0006 | 252 | 0.0135 | 0.0071 | -47.1731 | 0.0675 |
| **1970** | -0.0047 | -0.0098 | 0.0003 | 343 | 0.0124 | 0.0075 | -39.4240 | 0.0518 |
| **1971** | -0.0044 | -0.0095 | -0.0008 | 403 | 0.0115 | 0.0066 | -42.7406 | **0.0071** |
| **1972** | -0.0033 | -0.0073 | -0.0005 | 454 | 0.0086 | 0.0067 | -22.4284 | **0.0166** |
| **1973** | -0.0023 | -0.0064 | 0.0007 | 522 | 0.0073 | 0.0067 | -8.0511 | 0.0726 |
| **1974** | -0.0039 | -0.0100 | -0.0009 | 614 | 0.0091 | 0.0062 | -31.8228 | **0.0068** |
| **1975** | -0.0042 | -0.0089 | -0.0014 | 809 | 0.0081 | 0.0055 | -32.6215 | **0.0138** |
| **1976** | -0.0035 | -0.0066 | -0.0002 | 978 | 0.0079 | 0.0061 | -22.8913 | **0.0376** |
| **1977** | -0.0057 | -0.0094 | -0.0025 | 1228 | 0.0099 | 0.0065 | -33.7899 | **0.0004** |
| **1978** | -0.0053 | -0.0078 | -0.0029 | 1477 | 0.0102 | 0.0063 | -38.5432 | **0.0000** |
| **1979** | -0.0059 | -0.0088 | -0.0033 | 1719 | 0.0104 | 0.0063 | -39.9118 | **0.0000** |
| **1980** | -0.0050 | -0.0083 | -0.0022 | 1941 | 0.0104 | 0.0069 | -33.5579 | **0.0001** |
| **1981** | -0.0043 | -0.0075 | -0.0018 | 2109 | 0.0105 | 0.0065 | -38.3551 | **0.0003** |
| **1982** | -0.0033 | -0.0061 | -0.0011 | 2302 | 0.0101 | 0.0065 | -35.8162 | **0.0012** |
| **1983** | -0.0027 | -0.0043 | -0.0013 | 2876 | 0.0074 | 0.0043 | -41.4815 | **0.0003** |
| **1984** | -0.0024 | -0.0037 | -0.0012 | 3062 | 0.0079 | 0.0044 | -44.2713 | **0.0006** |
| **1985** | -0.0019 | -0.0031 | -0.0005 | 3138 | 0.0074 | 0.0043 | -41.0827 | **0.0063** |
| **1986** | -0.0018 | -0.0029 | -0.0009 | 3197 | 0.0070 | 0.0044 | -36.8396 | **0.0007** |
| **1987** | -0.0013 | -0.0022 | -0.0003 | 3187 | 0.0067 | 0.0044 | -34.7750 | **0.0051** |
| **1988** | -0.0012 | -0.0021 | -0.0002 | 3072 | 0.0063 | 0.0045 | -27.6112 | **0.0121** |
| **1989** | -0.0014 | -0.0025 | -0.0005 | 2916 | 0.0063 | 0.0041 | -33.8633 | **0.0017** |

| Breakpoint year | median | lower CI | upper CI | *n* | median before breakpoint year | median since breakpoint year | percentage change | p-value |
|---|---|---|---|---|---|---|---|---|
| **1990** | -0.0019 | -0.0031 | -0.0010 | 2672 | 0.0063 | 0.0040 | -35.5074 | **0.0000** |
| **1991** | -0.0013 | -0.0019 | -0.0004 | 2407 | 0.0057 | 0.0040 | -29.6388 | **0.0049** |
| **1992** | -0.0012 | -0.0020 | -0.0002 | 2674 | 0.0048 | 0.0036 | -24.9628 | **0.0261** |
| **1993** | -0.0017 | -0.0026 | -0.0007 | 3019 | 0.0046 | 0.0039 | -14.6152 | **0.0007** |
| **1994** | -0.0016 | -0.0027 | -0.0009 | 3177 | 0.0047 | 0.0038 | -19.0947 | **0.0002** |
| **1995** | -0.0014 | -0.0024 | -0.0008 | 3264 | 0.0046 | 0.0038 | -17.1064 | **0.0003** |
| **1996** | -0.0020 | -0.0028 | -0.0009 | 3274 | 0.0051 | 0.0035 | -30.6656 | **0.0002** |
| **1997** | -0.0023 | -0.0033 | -0.0012 | 4808 | 0.0054 | 0.0038 | -30.1519 | **0.0000** |
| **1998** | -0.0014 | -0.0024 | -0.0006 | 5799 | 0.0059 | 0.0054 | -7.8849 | **0.0004** |
| **1999** | -0.0017 | -0.0027 | -0.0008 | 6051 | 0.0062 | 0.0051 | -18.7049 | **0.0001** |
| **2000** | -0.0009 | -0.0018 | 0.0002 | 5748 | 0.0061 | 0.0053 | -12.8338 | 0.0794 |
| **2001** | -0.0012 | -0.0024 | 0.0000 | 4152 | 0.0048 | 0.0051 | 6.6182 | **0.0487** |
| **2002** | -0.0019 | -0.0032 | -0.0004 | 2335 | 0.0053 | 0.0049 | -6.7068 | **0.0116** |
| **2003** | -0.0043 | -0.0058 | -0.0029 | 1890 | 0.0071 | 0.0065 | -8.6839 | **0.0000** |
| **2004** | -0.0114 | -0.0150 | -0.0081 | 1230 | 0.0161 | 0.0065 | -59.3742 | **0.0000** |
| **2005** | -0.0099 | -0.0148 | -0.0071 | 928 | 0.0195 | 0.0059 | -69.6173 | **0.0000** |
| **2006** | -0.0040 | -0.0063 | -0.0017 | 339 | 0.0108 | 0.0082 | -24.3119 | **0.0005** |
| **2007** | -0.0028 | -0.0039 | 0.0002 | 242 | 0.0089 | 0.0078 | -12.9272 | 0.0621 |
| **2008** | -0.0035 | -0.0055 | -0.0005 | 210 | 0.0079 | 0.0086 | 8.0959 | **0.0322** |
| **2009** | -0.0018 | -0.0055 | 0.0023 | 279 | 0.0082 | 0.0080 | -2.7637 | 0.3381 |
| **2010** | -0.0032 | -0.0060 | 0.0011 | 265 | 0.0092 | 0.0073 | -20.9046 | 0.1403 |
| **2011** | 0.0017 | -0.0027 | 0.0046 | 109 | 0.0063 | 0.0083 | 31.1227 | 0.2503 |
| **2012** | -0.0013 | -0.0189 | 0.0111 | 21 | 0.0165 | 0.0145 | -11.9781 | 1.0000 |
| **2013** | -0.0021 | -0.0199 | 0.0203 | 14 | 0.0173 | 0.0156 | -9.5018 | 1.0000 |
| **2014** | -0.0155 | -0.0299 | 0.0231 | 14 | 0.0183 | 0.0020 | -89.2440 | 0.1796 |

**Table S2.** Metadata of studies from the BioTIME database included in this study

| ID | Realm | Climate | Taxon | Title | Start | End |
|---|---|---|---|---|---|---|
| 18 | Terrestrial | Temperate | Plants | Mapped quadrats in sagebrush steppe long-term data for analyzing demographic rates and plant to plant interactions | 1923 | 1973 |
| 33 | Marine | Temperate | Plants | Long-term phytoplankton community dynamics in the Western English Channel | 1992 | 2009 |
| 39 | Terrestrial | Temperate | Birds | Bird community dynamics in a temperate deciduous forest Long-term trends at Hubbard Brook | 1970 | 2015 |
| 46 | Terrestrial | Temperate | Birds | Skokholm Bird Observatory | 1928 | 1979 |
| 47 | Terrestrial | Temperate | Birds | Detection of Density-Dependent Effects in Annual Duck Censuses | 1952 | 1977 |
| 51 | Terrestrial | Temperate | Birds | Fluctuations and long-term in the relative densities of tetraonid populations in Finland. 1964-77 | 1964 | 1977 |
| 52 | Terrestrial | Polar | Mammals | A transect survey of small land carnivore and red fox populations on a subarctic fell in Finnish forest Lapland over 13 winters | 1968 | 1980 |
| 53 | Terrestrial | Temperate | Mammals | An 11-year study of small mammal populations at Mont St. Hilaire. Quebec | 1966 | 1976 |
| 54 | Terrestrial | Tropical | Invertebrates | El Verde Grid invertebrate data (Big Grid Snail Captures 1991-2007) | 1991 | 2023 |
| 56 | Terrestrial | Temperate | Mammals | Small Mammal Mark-Recapture Population Dynamics at Core Research Sites | 1989 | 2019 |
| 58 | Terrestrial | Tropical | Birds | Avian populations long-term monitoring dataset. San Juan. Puerto Rico Luquillo Long Term Ecological Research Site Database Grid points bird counts DBAS 23 | 1991 | 2008 |
| 59 | Terrestrial | Temperate | Mammals | Long-term monitoring and experimental manipulation of a Chihuahuan Desert ecosystem near Portal. Arizona. USA | 1977 | 2002 |
| 67 | Terrestrial | Temperate | Birds | Animal Demography Unit - Coordinated Waterbird Counts (CWAC) (AfrOBIS) | 1983 | 2006 |
| 68 | Marine | Temperate | Invertebrates | Marine and Coastal Management - Copepod Surveys (AfrOBIS) | 1988 | 2000 |
| 78 | Marine | Temperate | Invertebrates | IOW Macrozoobenthos monitoring Baltic Sea (1980-2005) (EurOBIS) | 1980 | 2005 |
| 81 | Marine | Temperate | Mammals | CRRU Cetacean sighting in Scotland waters 1997-2010 | 1997 | 2010 |
| 86 | Marine | Temperate | Plants | Phytoplankton of the Ukrainian Black Sea shelf (1985-2005) | 1985 | 2007 |
| 87 | Marine | Temperate | Plants | Phytoplankton in the Oosterschelde before. during and after the storm-surge barrier (1982-1990) | 1982 | 1990 |
| 90 | Marine | Temperate | Invertebrates | Zooplankton in the Bay of Biscay (1995-2004. yearly DEPM surveys) | 1995 | 2004 |
| 91 | Marine | Temperate | Birds | Baltic seabirds transect surveys | 1992 | 1999 |
| 108 | Marine | Global | Birds | Seabirds of the Southern and South Indian Ocean (Australian Antarctic Data Centre) | 1981 | 2006 |
| 110 | Marine | Temperate | Invertebrates | Marine Nature Conservation Review (MNCR) and associated benthic marine data held and managed by JNCC (EurOBIS) | 1954 | 2000 |
| 119 | Marine | Temperate | Fish | DFO Maritimes Research Vessel Trawl Surveys Fish Observations (OBIS Canada) | 1970 | 2010 |

| ID | Realm | Climate | Taxon | Title | Start | End |
|---|---|---|---|---|---|---|
| 121 | Marine | Temperate/Tropical | Fish | CRED Rapid Ecological Assessments of Fish Belt Transect Surveys and Fish Stationary Point Count Surveys in the Pacific Ocean 2000-2010 (OBIS-USA) | 2000 | 2010 |
| 123 | Marine | Temperate | Fish | Maine Department of Marine Resources Inshore Trawl Survey 2000-2009 (OBIS-USA) | 2000 | 2009 |
| 125 | Marine | Temperate | Fish | MARMAP Chevron Trap Survey 1990-2009 (OBIS-USA) | 1988 | 2000 |
| 127 | Marine | Temperate | Fish | MARMAP Florida Antillean Trap Survey 1990-2009 (OBIS-USA) | 1980 | 1989 |
| 148 | Marine | Temperate/Tropical | Fish | South Western Pacific Regional OBIS Data provider for the NIWA Marine Biodata Information System (South Western Pacific OBIS) | 1964 | 2005 |
| 152 | Marine | Temperate | Invertebrates | CMarZ (Census of Marine Zooplankton)-Asia Database | 1977 | 2002 |
| 162 | Marine | Temperate/Tropical | Invertebrates | EPA'S EMAP Database | 1990 | 2004 |
| 163 | Marine | Temperate | Invertebrates | North Pacific Groundfish Observer (North Pacific Research Board) | 1993 | 2004 |
| 166 | Marine | Global | Mixed | PIROP Northwest Atlantic 1965-1992 (SEAMAP) | 1965 | 1992 |
| 169 | Marine | Temperate | Mixed | CalCOFI and NMFS Seabird and Marine Mammal Observation Data. 1987-2006 (SEAMAP) | 1987 | 2006 |
| 171 | Marine | Temperate/Tropical | Mammals | Bahamas Marine Mammal Research Organisation Opportunistic Sightings (SEAMAP) | 1988 | 2008 |
| 172 | Marine | Temperate | Mixed | POPA cetacean. seabird. and sea turtle sightings in the Azores area 1998-2009 (OBIS SEAMAP) | 1998 | 2009 |
| 176 | Marine | Temperate | Invertebrates | Atlantic Zone Monitoring Program Maritimes Region (AZMP) plankton datasets. In Fisheries and Oceans Canada - BioChem archive (OBIS Canada) | 1998 | 2010 |
| 178 | Marine | Temperate | Fish | Pacific Shrimp Trawl Survey (OBIS Canada) | 1963 | 2007 |
| 180 | Marine | Polar/Temperate | Fish | ECNASAP - East Coast North America Strategic Assessment (OBIS Canada) | 1970 | 1995 |
| 182 | Marine | Temperate | Mixed | Snow crab research trawl survey database (Southern Gulf of St. Lawrence. Gulf region. Canada) from 1988 to 2010 (OBIS Canada) | 1988 | 2009 |
| 183 | Marine | Temperate | Invertebrates | DFO Maritimes Research Vessel Trawl Surveys Invertebrate Observations (OBIS Canada) | 1999 | 2011 |
| 189 | Marine | Tropical | Fish | St. John. USVI Fish Assessment and Monitoring Data (2002 - Present) (NOAA-CCMA) | 2001 | 2010 |
| 190 | Marine | Tropical | Fish | St. Croix. USVI Fish Assessment and Monitoring Data (2002 - Present) (NOAA-CCMA) | 2001 | 2010 |
| 192 | Marine | Polar/Temperate | Mammals | Whale Catches in Southern Ocean (OBIS - Australian Antarctic Data Centre) | 1932 | 1980 |
| 194 | Terrestrial | Temperate | Invertebrates | Spatial and temporal distribution and abundance of moths in the Andrews Experimental Forest. 1994 to 2004 | 1994 | 2004 |
| 195 | Terrestrial | Temperate | Birds | Breeding birds survey North America | 1978 | 2007 |
| 196 | Marine | Temperate | Invertebrates | SOTEAG Rocky Shore Survey (Sullom Voe) | 1976 | 2012 |

| ID | Realm | Climate | Taxon | Title | Start | End |
|---|---|---|---|---|---|---|
| 204 | Marine | Temperate | Invertebrates | MACROBEL Long term trends in the macrobenthos of the Belgian Continental Shelf | 1976 | 2001 |
| 211 | Marine | Temperate | Fish | MARMAP Fly Net 1990-2009 | 1980 | 1987 |
| 213 | Marine | Temperate | Mixed | Northeast Fisheries Science Center Bottom Trawl Survey Data (OBIS-USA) | 1948 | 2008 |
| 214 | Terrestrial | Temperate | Plants | Long-term growth mortality and regeneration of trees in permanent vegetation plots in the Pacific Northwest 1910 to present | 1910 | 2010 |
| 217 | Terrestrial | Temperate | Birds | Landbird Monitoring Program (UMT-LBMP) | 1992 | 2006 |
| 221 | Terrestrial | Temperate | Plants | Vegetation Plots of the Bonanza Creek LTER Control Plots Species Count (1975 - 2004) | 1975 | 2008 |
| 225 | Terrestrial | Temperate | Birds | Point count bird censusing long-term monitoring of bird distrubution and diversity in central Arizona-Phoenix period 2000 to 2011 | 2000 | 2011 |
| 229 | Freshwater | Temperate | Fish | Upper Little Tennessee River Biomonitoring Program Database - LTWA Biomonitoring Database | 1988 | 2013 |
| 236 | Freshwater | Temperate | Fish | Fish population on selected watersheds at Konza Prairie - CFP012 - Konza fish population | 1995 | 2006 |
| 237 | Freshwater | Temperate | Invertebrates | Madison Wisconsin Lakes Zooplankton 1976 - 1994 - old net | 1976 | 1994 |
| 238 | Freshwater | Temperate | Invertebrates | North Temperate Lakes LTER Zooplankton - Madison Lakes Area 1997 - current | 1995 | 2014 |
| 240 | Terrestrial | Temperate | Plants | Pinon-Juniper (Core Site) Quadrat Data for the Net Primary Production Study at the Sevilleta National Wildlife Refuge New Mexico (2003-present ) | 2003 | 2015 |
| 242 | Terrestrial | Temperate | Plants | Lac Croche understory vegetation data set (1998 to 2006) | 1998 | 2006 |
| 243 | Terrestrial | Temperate | Plants | Long-term N-fertilized vegetation plots on Hog Island Virginia Coastal Barrier Islands 1992 to 2014 | 1992 | 2014 |
| 246 | Marine | Temperate | Fish | Long-term monitoring dataset of fish assemblages impinged at nuclear power plants in northern Taiwan | 2000 | 2014 |
| 248 | Terrestrial | Temperate | Plants | Evidence for long-term shift in plant community composition under decadal experimental warming | 2000 | 2012 |
| 252 | Marine | Temperate | Fish | MARMAP Blackfish Trap Survey 1990-2009 | 1977 | 1989 |
| 255 | Terrestrial | Temperate | Plants | Multi-decade, spatially explicit population studies of canopy dynamics in Michigan old-growth forests | 1993 | 2007 |
| 271 | Marine | Temperate | Fish | Santa Barbara Coastal LTER Reef: Kelp Forest Community Dynamics: Abundance and size of Giant Kelp (Macrocystis Pyrifera), ongoing since 2000 | 2000 | 2014 |
| 272 | Marine | Temperate | Invertebrates | Santa Barbara Coastal LTER Reef: Kelp Forest Community Dynamics: Fish abundance | 2000 | 2014 |
| 273 | Marine | Temperate | Invertebrates | Santa Barbara Coastal LTER Reef: Kelp Forest Community Dynamics: Invertebrate and algal density | 2000 | 2014 |

| ID | Realm | Climate | Taxon | Title | Start | End |
|---|---|---|---|---|---|---|
| 274 | Marine | Temperate | Mixed | Santa Barbara Coastal LTER Reef: Kelp Forest Community Dynamics: Cover of sessile organisms, Uniform Point Contact | 2000 | 2014 |
| 278 | Marine | Temperate/Tropical | Fish | South Western Pacific Regional OBIS Data provider for the NIWA Marine Biodata Information System (South Western Pacific OBIS) | 1961 | 2005 |
| 286 | Marine | Temperate | Fish | Maine Department of Marine Resources Inshore Trawl Survey 2000-2009 (OBIS-USA) | 2000 | 2009 |
| 288 | Marine | Temperate | Fish | DFO Maritimes Research Vessel Trawl Surveys Fish Observations (OBIS Canada) | 1970 | 2006 |
| 294 | Terrestrial | Tropical | Invertebrates | Tam Dao Butterfly communities | 2003 | 2013 |
| 295 | Marine | Temperate | Fish | Systematic global assessment of reef fish communities by the Reef Life Survey program | 2008 | 2016 |
| 296 | Marine | Temperate | Invertebrates | Systematic global assessment of reef fish communities by the Reef Life Survey program | 2008 | 2016 |
| 298 | Terrestrial | Temperate | Plants | Long-term mapped quadrats from Kansas prairie demographic information for herbaceaous plants | 1903 | 1972 |
| 300 | Terrestrial | Temperate | Invertebrates | Insect Populations via Sticky Traps at KBS-LTER (Kellogg Biological Station. MI) | 1989 | 2013 |
| 305 | Terrestrial | Temperate | Amphibians | Population estimates of Appalachian salamanders. Coweeta LTER | 1976 | 1990 |
| 306 | Freshwater | Temperate | Reptiles | Edwin S. George Reserve Turtles. The Global Population Dynamics Database Version 2 | 1975 | 1992 |
| 310 | Marine | Temperate/Tropical | Invertebrates | Marine microplankton diversity database | 1992 | 2002 |
| 311 | Terrestrial | Temperate | Mammals | Seasonal summary of numbers of small mammals on 14 LTER traplines in prairie habitats at Konza Prairie | 1981 | 2013 |
| 312 | Terrestrial | Tropical | Mammals | Stability in a Multi-Species Assemblage of Large Herbivores in East Africa | 1959 | 1984 |
| 313 | Terrestrial | Temperate | Invertebrates | Successional Dynamics on a Resampled Chronosequence Core Old Field Grasshopper Sampling | 1989 | 2006 |
| 317 | Terrestrial | Temperate | Plants | Mangrove Forest Growth from the Shark River Slough Everglades National Park (FCE) South Florida from January 1995 to Present | 1995 | 2005 |
| 319 | Terrestrial | Temperate | Mixed | Effects of rangeland management on community dynamics of herpetofauna to the tallgrass prairie | 1989 | 2003 |
| 321 | Terrestrial | Temperate | Mammals | Small Mammal Exclosure Study. Jornada LTER. SMES rodent trapping data | 1995 | 2007 |
| 327 | Terrestrial | Temperate | Mammals | Fray Jorge Small Mammals 1989-2005 | 1989 | 2005 |
| 328 | Freshwater | Temperate | Amphibians | The Rainbow Bay Long-term Study | 1979 | 2008 |
| 329 | Terrestrial | Tropical | Plants | Twenty years tree demography in an undisturbed Dipterocarp permanent sample plot at Uppangala Western Ghats of India | 1990 | 2010 |
| 330 | Marine | Tropical | Invertebrates | The Australian Zooplankton Database (Project ID 1): PhD; zooplankton abundance in Gulf of Carpentaria | 1975 | 1977 |

| ID | Realm | Climate | Taxon | Title | Start | End |
|---|---|---|---|---|---|---|
| 332 | Freshwater | Temperate | Fish | Stream Fish Assemblage stability in a southern Appalachian stream (Coweeta Hydro Lab 1984 - 1995) | 1984 | 1995 |
| 333 | Terrestrial | Temperate | Birds | Weekly record of bird species observed on Konza Prairie | 1981 | 2009 |
| 335 | Freshwater | Temperate | Fish | Stochasticity in structural and functional characteristics of an Indiana stream fish assemblage  a test of community theory | 1962 | 1974 |
| 336 | Terrestrial | Temperate | Plants | Long term monitoring and experimental manipulation of a Chihuahuan Desert ecosystem near Portal Arizona | 1989 | 2002 |
| 337 | Terrestrial | Temperate | Birds | Mountain Birdwatch | 2000 | 2010 |
| 339 | Terrestrial | Temperate | Birds | Species trends turnover and composition of a woodland bird community in southern Sweden during a period of 57 years. | 1953 | 2009 |
| 348 | Terrestrial | Temperate/Tropical | Mammals | Bats (Mammalia Chiroptera) in restinga in the municipality of Jaguaruna south of Santa Catarina Brazil. | 2006 | 2016 |
| 350 | Marine | Temperate | Invertebrates | Megafauna PAP time series | 1989 | 2011 |
| 351 | Marine | Temperate | Invertebrates | Megafauna Sta M time series | 1989 | 2004 |
| 355 | Terrestrial | Temperate | Plants | Plant Species Composition on Selected Watersheds at Konza Prairie | 1983 | 2015 |
| 356 | Terrestrial | Tropical | Plants | Long-term stem inventory data from tropical rain forest plots in Australia | 1971 | 2013 |
| 357 | Terrestrial | Temperate | Mammals | Small Mammal Trapping Webs on the Central Plains Experimental Range | 1994 | 2006 |
| 358 | Terrestrial | Temperate | Birds | Neotropical Migratory Bird Communities in a Developing Pine Plantation | 1977 | 1992 |
| 359 | Marine | Temperate | Fish | SBC LTER Reef Kelp Forest Community Dynamics Fish abundance | 2000 | 2012 |
| 360 | Terrestrial | Temperate | Birds | Bialowieza National Park bird assemblage | 1975 | 2014 |
| 361 | Terrestrial | Temperate | Birds | A long-term bird population study in an Appalachian spruce forest | 1962 | 1983 |
| 363 | Terrestrial | Temperate | Birds | The 37-year dynamics of a subalpine bird community with special emphasis on the influence of environmental temperature and Epirrita autumnata cycles. | 1963 | 1999 |
| 364 | Freshwater | Temperate | Plants | Long-term reductions in anthropogenic nutrients link to improvements in Chesapeake Bay habitat | 1990 | 2007 |
| 366 | Terrestrial | Temperate | Mammals | Small Mammal Exclosure Study (SMES) | 1989 | 2013 |
| 372 | Terrestrial | Temperate | Birds | Monitoring site 1000 Village survey - Bird survey data | 2005 | 2013 |
| 373 | Terrestrial | Temperate | Mammals | Village survey Medium and large mammal survey data | 2005 | 2012 |
| 374 | Marine | Temperate | Birds | Monitoring site 1000 Shorebird Survey | 2004 | 2014 |
| 375 | Terrestrial | Temperate | Invertebrates | Surface of the earth wandering beetles survey data | 2004 | 2014 |
| 378 | Marine | Temperate | Invertebrates | Calafuria Mid-shore Intertidal Dataset (1991-2014) | 1991 | 2006 |
| 379 | Marine | Temperate | Invertebrates | Calafuria Low-shore Intertidal Dataset (1991-2014) | 1991 | 2014 |
| 382 | Terrestrial | Temperate | Mammals | Small Mammals and Vegetation Changes After Fire in a Mixed Conifer-Hardwood Forest | 1955 | 1967 |
| 413 | Terrestrial | Temperate | Birds | Bird populations in east central Illinois. Fluctuations variations and development over a half-century | 1927 | 1976 |

| ID | Realm | Climate | Taxon | Title | Start | End |
|---|---|---|---|---|---|---|
| 414 | Terrestrial | Temperate | Birds | Bird populations in east central Illinois. Fluctuations variations and development over a half-century | 1924 | 1976 |
| 415 | Terrestrial | Temperate | Birds | Bird populations in east central Illinois. Fluctuations variations and development over a half-century | 1949 | 1967 |
| 416 | Terrestrial | Temperate | Birds | Bird populations in east central Illinois. Fluctuations variations and development over a half-century | 1946 | 1971 |
| 419 | Marine | Temperate | Birds | Data collected aboard cruises off the coast of the Western Antarctic Penninsula | 1993 | 2011 |
| 420 | Terrestrial | Polar/Temperate | Birds | Species composition and population fluctuations of alpine bird communities during 38 years in the Scandinavian mountain range | 1964 | 2001 |
| 426 | Freshwater | Temperate | Invertebrates | Phytoplankton time series from Lake Zurich | 1976 | 2010 |
| 427 | Freshwater | Temperate | Invertebrates | Zooplankton time series from Lake Zurich | 1977 | 2008 |
| 428 | Marine | Temperate | Mixed | Long term monitoring of fish abundances from coastal Skagerrak | 1919 | 2015 |
| 430 | Freshwater | Temperate | Fish | The New Zealand Freshwater Fish Database - Electric fishing - Backpack | 1985 | 2016 |
| 431 | Freshwater | Temperate | Fish | The New Zealand Freshwater Fish Database - Traps- Gee Minnow traps | 1984 | 2016 |
| 432 | Freshwater | Temperate | Fish | The New Zealand Freshwater Fish Database - Observation (Spotlighting visual) | 1998 | 2016 |
| 433 | Freshwater | Polar | Invertebrates | Zooplankton density for lake samples collected near Toolik Lake Arctic LTER in the summers (1983-1992 & 1993-2002) | 1983 | 2002 |
| 434 | Freshwater | Polar | Invertebrates | Summer Phytoplankton Densities 1992-2001 | 1992 | 2001 |
| 438 | Marine | Tropical | Fish | Aceh WCS fish surveys | 2006 | 2014 |
| 439 | Terrestrial | Temperate | Birds | Long-term dynamics of bird populations in pine forests of Ilmen Nature Reserve during the breeding period individuals / km2 | 1985 | 1997 |
| 440 | Terrestrial | Temperate | Birds | Long-term dynamics of bird populations in pine-birch forests of Ilmen Nature Reserve during the breeding period individuals / km2 | 1985 | 1997 |
| 441 | Terrestrial | Temperate | Birds | Long-term dynamics of bird populations in birch forests of Ilmen Nature Reserve during the breeding period individuals / km2 | 1985 | 1997 |
| 447 | Terrestrial | Temperate | Mammals | Long-term population dynamics of small mammals in the Natural Boundary Morozova Gora (individuals / 100 trap-nights) | 2006 | 2014 |
| 449 | Terrestrial | Temperate | Mammals | Indicators of abundance (individuals / 100 trap-nights) of different species of small mammals in different years with using trap grooves and a coefficient characterizing the adverse conditions winter | 2000 | 2009 |
| 452 | Marine | Temperate | Fish | Year-to-year dynamics of total nekton biomass (thous. tons) in the upper epipelagic of the North-West Pacific in June-July of 2000s | 2004 | 2012 |
| 456 | Marine | Tropical | Invertebrates | Dynamics of a coral reef community | 2003 | 2010 |
| 457 | Marine | Tropical | Invertebrates | Dynamics of a coral reef community | 2003 | 2014 |
| 466 | Marine | Temperate | Fish | Trawl Survey Data from Rockall Scotland (1986 - 2008) | 1986 | 2008 |

| ID | Realm | Climate | Taxon | Title | Start | End |
|---|---|---|---|---|---|---|
| 468 | Marine | Temperate | Invertebrates | A multi-decade time series of kelp forest community structure at San Nicolas Island California. | 1980 | 2011 |
| 469 | Marine | Temperate | Invertebrates | A multi-decade time series of kelp forest community structure at the California Channel Islands. | 1982 | 2011 |
| 471 | Terrestrial | Temperate | Plants | Prescribed Burn Effect on Chihuahuan Desert Grasses and Shrubs at the Sevilleta National Wildlife Refuge | 2004 | 2013 |
| 473 | Terrestrial | Temperate | Plants | Fourteen years of mapped permanent quadrats in a northern mixed prairie | 1932 | 1945 |
| 475 | Terrestrial | Temperate | Birds | Structure and dynamics of a passerine bird community in a spruce-dominated boreal forest | 1960 | 1972 |
| 477 | Marine | Temperate | Invertebrates | Epifaunal invertebrate survey from Goodwin Islands York River Estuary Chesapeake Bay | 1998 | 2012 |
| 478 | Freshwater | Temperate | Invertebrates | Long term study of the stream ecosystems in the Breitenbach | 1969 | 2005 |
| 479 | Terrestrial | Polar | Plants | ITEX Dataset 1 - Abisko (Wet, Dry, Peatland), Kanger (Bashful, Dopey, Sneezy) and Kilpisjarvi | 1999 | 2008 |
| 483 | Terrestrial | Temperate | Plants | ITEX Dataset 5 - Teberda (Malaya Alpine-Snowbed and Geranium Hedysarum Meadow) | 1986 | 2009 |
| 491 | Terrestrial | Polar | Plants | ITEX Dataset 13 - Toolik (Dry, Moist) | 1994 | 2008 |
| 492 | Terrestrial | Polar | Plants | ITEX Dataset 14 - Toolik (LTER Heath, LTER Moist acidic tussock, LTER non-acidic tussock, LTER wet sedge, SAG wet sedge2, Tussock 1981 plots) | 1982 | 2006 |
| 497 | Terrestrial | Temperate | Plants | ITEX Dataset 19 - Teberda (Festuca Varia Grassland, Malaya Alpine Lichen-Heath) | 1981 | 2009 |
| 499 | Marine | Temperate | Invertebrates | Macrobenthos monitoring at long-term monitoring stations in the Belgian part of the North Sea between 1979 and 1999 | 1979 | 1999 |
| 500 | Marine | Temperate | Invertebrates | Macrobenthos monitoring at long-term monitoring stations in the Belgian part of the North Sea from 2001 on | 2001 | 2012 |
| 501 | Marine | Temperate | Invertebrates | Epibenthos and demersal fish monitoring at long-term monitoring stations in the Belgian part of the North Sea (Trawl Distance 3000m) | 2004 | 2012 |
| 505 | Marine | Temperate | Mixed | Fish and marine invertebrates from the Israeli Eastern Mediterranean sea 1990-4, 2000, 2008-2012 | 1990 | 2012 |
| 507 | Marine | Temperate | Fish | Trawl fisheries in Israeli Mediterranean | 1976 | 2010 |
| 509 | Terrestrial | Polar | Plants | ITEX Dataset 22 - Toolik (Imnavait, Tussockgrid) | 1989 | 2008 |
| 511 | Marine | Tropical | Fish | Demersal fish hauls from Guanabara Bay Brazil 2005-2015, first period | 2005 | 2007 |
| 380 | Terrestrial | Temperate | Invertebrates | Monitoring butterfly numbers | 1978 | 1987 |
| 63 | Terrestrial | Temperate | Invertebrates | Monitoring butterfly numbersThe development of dragonfly communities and the consequences of territorial behaviour A 27-year study on small ponds at Woodwalton Fen. Cambridgeshire. United Kingdom | 1962 | 1988 |

| ID | Realm | Climate | Taxon | Title | Start | End |
|---|---|---|---|---|---|---|
| 301 | Terrestrial | Temperate | Invertebrates | Konza Prarie grasshoppers - CGR022 | 1982 | 2018 |
| 57 | Freshwater | Temperate | Fish | North Temperate Lakes LTER Fish Abundance - Crayfish traps | 1981 | 2021 |
| 232 | Marine | Polar/Temperate | Fish | Pelagic Fish Observations 1968-1999 | 1968 | 1999 |
| 247 | Freshwater | Temperate | Invertebrates | Zooplankton survey of Oneida Lake New York 1964 to present | 1975 | 2020 |
| 249 | Terrestrial | Temperate | Invertebrates | Resource specialists lead local insect community turnover associated with temperature - analysis of an 18-year full-seasonal record of moths and beetles | 1994 | 2009 |
| 253 | Freshwater | Temperate | Invertebrates | North Temperate Lakes LTER Zooplankton - Trout Lake Area 1982 - current | 1981 | 2020 |
| 254 | Freshwater | Temperate | Plants | North Temperate Lakes LTER: Phytoplankton - Madison Lakes Area 1995 - current | 1995 | 2020 |
| 297 | Marine | Tropical | Invertebrates | MCR LTERCoral Reef Long-term Population and Community Dynamics Other Benthic Invertebrates. ongoing since 2005 | 2005 | 2022 |
| 334 | Terrestrial | Polar | Plants | Above ground plant biomass in a mesic acidic tussock tundra experimental site from 1982 to 2015 Arctic LTER, Toolik Lake, Alaska | 1982 | 2000 |
| 340 | Terrestrial | Tropical | Plants | Small Mammal Exclosure Study (SMES) Vegetation Data from the Chihuahuan Desert Grassland and Shrubland at the Sevilleta National Wildlife Refuge, New Mexico (1995-2009) | 1995 | 2009 |
| 354 | Marine | Polar/Temperate | Invertebrates | The Australian Phytoplankton Database (1844 - ongoing): IMOS Australian Continuous Plankton Recorder Survey | 2007 | 2016 |
| 99 | Marine | Temperate/Tropical | Fish | CSIRO, fish surveys by the Courageous, 1978-1979 | 1978 | 1979 |
| 308 | Terrestrial | Temperate | Mammals | Long Term Mammal Data from Powdermill Biological Station 1979-1999 | 1979 | 1999 |
| 435 | Marine | Polar/Temperate | Mixed | Zooplankton collected with a 2-m, 700-um net towed from surface to 120 m, aboard Palmer Station Antarctica LTER annual cruises off the western Antarctic peninsula, 2009 - 2020 | 2009 | 2020 |
| 191 | Marine | Temperate | Invertebrates | NEFSC Benthic Database (OBIS-USA) - Otter Trawl | 1953 | 1975 |
| 200 | Marine | Temperate | Invertebrates | NEFSC Benthic Database (OBIS-USA) - Smith McIntyre grabs | 1956 | 1989 |

**Table S3:** Details of the 23 studies that were included in the 1975 breakyear turnover rate analysis

| STUDY_ID | REALM | CLIMATE | BIOME_MAP | TAXA | ORGANISMS | TITLE | START YEAR | END YEAR | CENTRAL LATITUDE | CENTRAL LONGITUDE | NUMBER of SPECIES | NUMBER of SAMPLES | SUMMARY of METHODS |
|---|---|---|---|---|---|---|---|---|---|---|---|---|---|
| 39 | Terrestrial | Temperate | Temperate broadleaf and mixed forests | Birds | birds | Bird community dynamics in a temperate deciduous forest Long-term trends at Hubbard Brook | 1970 | 2015 | 43.91 | -71.75 | 52 | 45 | Plots |
| 46 | Terrestrial | Temperate | Temperate broadleaf and mixed forests | Birds | breeding bird pairs | Skokholm Bird Observatory | 1928 | 1979 | 51.698 | -5.277 | 29 | 47 | Counts within defined area |
| 63 | Terrestrial | Temperate | Small lake ecosystems | Terrestrial invertebrates | dragonflies | The development of dragonfly communities and the consequences of territorial behaviour A 27-year study on small ponds at Woodwalton Fen. Cambridgeshire. United Kingdom | 1959 | 1988 | 52.4221 | -0.180928 | 5 | 1 | Counts within defined area |
| 119 | Marine | Temperate | Temperate shelf and seas ecoregions | Fish | fish | DFO Maritimes Research Vessel Trawl Surveys Fish Observations (OBIS Canada) | 1970 | 2010 | 43.987425 | -63.669701 | 231 | 13945 | Transects |
| 148 | Marine | Temperate/Tropical | Temperate shelf and seas ecoregions | Fish | fish | South Western Pacific Regional OBIS Data provider for the NIWA Marine Biodata Information System (South Western Pacific OBIS) | 1964 | 2005 | -42.407 | 172.26 | 430 | 28775 | Stations |
| 166 | Marine | Global | Multiple ecoregions | Mixed | pelagic seabirds | PIROP Northwest Atlantic 1965-1992 (SEAMAP) | 1965 | 1992 | 36.075242 | -70.991806 | 213 | 144510 | Surveys |
| 178 | Marine | Temperate | Temperate shelf and seas ecoregions | Fish | mostly fish + some marine invertebrates | Pacific Shrimp Trawl Survey (OBIS Canada) | 1963 | 2007 | 53.54327 | -140.266034 | 476 | 8065 | Transects |
| 180 | Marine | Polar/Temperate | Polar ecoregions | Fish | groundfish | ECNASAP - East Coast North America Strategic Assessment (OBIS Canada) | 1970 | 1995 | 37.770564 | -50.792666 | 273 | 46466 | Trawl |
| 192 | Marine | Polar/Temperate | Polar ecoregions | Mammals | whales | Whale Catches in Southern Ocean (OBIS - Australian Antarctic Data Centre) | 1932 | 1980 | -62.076411 | 79.717188 | 6 | 455 | Transects |
| 200 | Marine | Temperate | Temperate shelf and seas ecoregions | Marine invertebrates | marine invertebrates | NEFSC Benthic Database (OBIS-USA) | 1956 | 1989 | 35.691706 | -74.090761 | 2105 | 2945 | Transects |
| 213 | Marine | Temperate | Temperate shelf and seas ecoregions | Benthos | benthos | Northeast Fisheries Science Center Bottom Trawl Survey Data (OBIS-USA) | 1948 | 2008 | 36.625131 | -72.635974 | 1023 | 35644 | Bottom trawl |
| 214 | Terrestrial | Temperate | Temperate coniferous forest | Terrestrial plants | trees | Long-term growth mortality and regeneration of trees in permanent vegetation plots in the Pacific Northwest 1910 to present | 1910 | 2010 | 45.34296 | -122.799 | 39 | 14565 | Plots |
| 232 | Marine | Polar/Temperate | Polar ecoregions | Fish | fish | Pelagic Fish Observations 1968-1999 | 1968 | 1999 | -56.814716 | 93.88436 | 185 | 3143 | Voyage data |
| 278 | Marine | Temperate/Tropical | Temperate shelf and seas ecoregions | Fish | fish | South Western Pacific Regional OBIS Data provider for the NIWA Marine Biodata Information System (South Western Pacific OBIS) | 1961 | 2005 | -42.407 | 172.26 | 278 | 5177 | Stations |
| 288 | Marine | Temperate | Temperate shelf and seas ecoregions | Fish | fish | DFO Maritimes Research Vessel Trawl Surveys Fish Observations (OBIS Canada) | 1970 | 2006 | 43.977389 | -63.682015 | 195 | 6774 | Transects |
| 312 | Terrestrial | Tropical | Tropical and subtropical grasslands, savannas and shrublands | Mammals | large herbivores | Stability in a Multi-Species Assemblage of Large Herbivores in East Africa | 1959 | 1984 | 3.5 | 35.75 | 13 | 9 | Census |
| 330 | Marine | Temperate/Tropical | Multiple ecoregions | Marine invertebrates | zooplanKton | Over 75 years of zooplankton data from Australia | 1938 | 2014 | -23.830553 | 136.449216 | 639 | 4179 | Nets |
| 339 | Terrestrial | Temperate | Temperate broadleaf and mixed forests | Birds | birds | Species trends turnover and composition of a woodland bird community in southern Sweden during a period of 57 years. | 1953 | 2009 | 55.71667 | 13.33333 | 39 | 57 | Grids |
| 361 | Terrestrial | Temperate | Temperate broadleaf and mixed [illegible] | Birds | birds | A long-term bird population study in an Appalachian spruce forest | 1962 | 1983 | 38.61 | -79.83472 | 20 | 22 | Mark_recapture |
| 363 | Terrestrial | Temperate | Tundra | Birds | birds | The 37-year dynamics of a subalpine bird community with special emphasis on the influence of environmental temperature and Epirrita autumnata cycles. | 1963 | 1999 | 65.968055 | 16.31666 | 35 | 37 | Ground data |
| 420 | Terrestrial | Polar/Temperate | Tundra | Birds | birds | Species composition and population fluctuations of alpine bird communities during 38 years in the Scandinavian mountain range | 1964 | 2001 | 67.077 | 17.435 | 47 | 99 | Sites |
| 428 | Marine | Temperate | Temperate shelf and seas ecoregions | Mixed | mainly fish and some invertebrates | Long term monitoring of fish abundances from coastal SKagerrak | 1919 | 2015 | 58.95856 | 9.768152 | 59 | 10725 | Seine nets |
| 478 | Freshwater | Temperate | Small river ecosystems | Freshwater invertebrates | insects | Long term study of the stream ecosystems in the Breitenbach | 1969 | 2005 | 50.669722 | 9.628889 | 90 | 37 | Nets |

# Supplementary Note 1

## Comparison of robustness of the Sørensen and Ochiai similarity metrics to false negative errors

We consider similarity metrics computed from three different survey years labeled a, b, c . For simplicity, we assume that species lost between a and b do not re-emerge in year c (however, the appearance of such re-emergence is possible due to false negatives in year b ) .
This leads to 5 groups of species depending on the years in which they are observed. We denote the numbers of species in these groups by n1, ..., n5 as follows:

```
n1 n2 n3 00 00 present in year a
00 n2 n3 n4 00 present in year b
00 00 n3 n4 n5 present in year c
```

For example, there are n5 species present only in year c and n3 species present in all three years a, b, and c. Due to false negatives, not all species are observed in all years.

We denote the number of observations from the different groups in each year using the coding o<group index><year label>, which leads to the following numbers observed in each year:

```
o1a o2a o3a 000 000 observed in year a
000 o2b o3b o4b 000 observed in year b
000 000 o3c o4c o5c observed in year c
```

We denote the size of the set of species in group 2 observed in both years a and b by j2ab, and of the species in group 3 the size of the set jointly observed in years a and b by j3ab and of those jointly observed in years a and c by j3ac:

```
0000 j2ab j3ab 0000 0000 = jointly observed in a and b
0000 0000 j3ac 0000 0000 = jointly observed in a and c
```

We use the notation S < year label > < year label > to denote Sørensen similarity indices and O < year label > < year label > to denote Ochiai similarity indices. Thus:

```
In[∘]:= Sab = 2 (j2ab + j3ab) / (o1a + o2a + o3a + o2b + o3b + o4b);
Sac = 2 (j3ac) / (o1a + o2a + o3a + o3c + o4c + o5c);
Oab = (j2ab + j3ab) / Sqrt[(o1a + o2a + o3a) (o2b + o3b + o4b)];
Oac = (j3ac) / Sqrt[(o1a + o2a + o3a) (o3c + o4c + o5c)];
```

We'd like to determine the statistical properties of the change in similarity with year a between years b and c, for both the Sørensen and the Ochiai metric, i.e., of:

In[∘]:=
```
Sd = Sab - Sac // FullSimplify (* change in similarity (Sørensen) *)
Od = Oab - Oac // FullSimplify(* change in similarity (Ochiai) *)
```

Out[∘]= $$\frac{2\,(j2ab + j3ab)}{o1a + o2a + o2b + o3a + o3b + o4b} - \frac{2\,j3ac}{o1a + o2a + o3a + o3c + o4c + o5c}$$

Out[∘]= $$\frac{j2ab + j3ab}{\sqrt{(o1a + o2a + o3a)\,(o2b + o3b + o4b)}} - \frac{j3ac}{\sqrt{(o1a + o2a + o3a)\,(o3c + o4c + o5c)}}$$

In these formulas, the following are random variates:

In[∘]:=
```
variables = Union@Cases[{Sd, Od}, x_?AtomQ /; Not[NumberQ[x]] :> x, {0, -1}]
```

Out[∘]= {j2ab, j3ab, j3ac, o1a, o2a, o2b, o3a, o3b, o3c, o4b, o4c, o5c}

To simplify the problem further and so reduce the number of parameters and the size of the resulting formulas, we assume that in each of the three years the same number of species, n, is present (the number of observed species may vary). This allows us to eliminate n3, n4, and n5 using the following rule:

In[∘]:=
```
nRules = Solve[n1 + n2 + n3 == n && n2 + n3 + n4 == n && n3 + n4 + n5 == n, {n3, n4, n5}] // Last
```

Out[∘]= {n3 → n - n1 - n2, n4 → n1, n5 → n2}

We assume that there are species in each of the five groups. Doing so, we can exclude some special cases in the calculations below. The final results might well be valid if some of the groups are empty. The following expresses this assumption in terms of n, n1, and n2:

In[∘]:=
```
$Assumptions =.
$Assumptions = n1 > 0 && n2 > 0 && n3 > 0 && n4 > 0 && n5 > 0 && n > 0 /. nRules // FullSimplify
```

Out[∘]= n1 > 0 && n > n1 + n2 && n2 > 0

We assume that for a species that is present in a year the probability of observing it in a survey is $\omega$. Correspondingly, the false negative rate is 1-$\omega$.

On this basis, we now construct a function that computes expectation values of expressions containing the random variates identified above. This is taking into account that the distribution of the size of the sets of species jointly observed in two years depends on the numbers of species separately observed in each year:

In[∘]:=
```
innerExpectation[x_] := If[FreeQ[x, j2ab | j3ab | j3ac], x,
  Expectation[x,
   {j2ab ≈ HypergeometricDistribution[o2a, o2b, n2],
    j3ab ≈ HypergeometricDistribution[o3a, o3b, n3],
    j3ac ≈ HypergeometricDistribution[o3a, o3c, n3]}]]
expectation[x_] :=
 Assuming[$Assumptions && 0 < ω < 1,
  Expectation[innerExpectation[x],
    {o1a ≈ BinomialDistribution[n1, ω], o2a ≈ BinomialDistribution[n2, ω],
     o2b ≈ BinomialDistribution[n2, ω], o2c ≈ BinomialDistribution[n2, ω],
     o3a ≈ BinomialDistribution[n3, ω], o3b ≈ BinomialDistribution[n3, ω],
     o3c ≈ BinomialDistribution[n3, ω], o4b ≈ BinomialDistribution[n4, ω],
     o4c ≈ BinomialDistribution[n4, ω], o5c ≈ BinomialDistribution[n5, ω]}
   ] /. nRules]
```

Here is an example of how this works:

In[∘]:=
```
(* Examples *)
innerExpectation[j3ac]
expectation[j3ac]
```

Out[∘]= $\frac{\text{o3a o3c}}{\text{n3}}$

Out[∘]= $(\text{n} - \text{n1} - \text{n2})\,\omega^2$

We now compute for each of the random variates its expectation value for later use and denote it by EE[<variate name>]:

In[∘]:=
```
For[i = 1, i ≤ Length[variables], i++,
 EE[variables[[i]] // Evaluate] = expectation[variables[[i]]]
]
? EE
```

Out[∘]=

Symbol

Global`EE

Definitions

EE[j2ab] = n2 $\omega^2$

EE[j3ab] = (n – n1 – n2) $\omega^2$

EE[j3ac] = (n – n1 – n2) $\omega^2$

EE[o1a] = n1 $\omega$

EE[o2a] = n2 $\omega$

EE[o2b] = n2 $\omega$

EE[o3a] = (n – n1 – n2) $\omega$

EE[o3b] = (n – n1 – n2) $\omega$

EE[o3c] = (n – n1 – n2) $\omega$

EE[o4b] = n1 $\omega$

EE[o4c] = n1 $\omega$

EE[o5c] = n2 $\omega$

Full Name Global`EE

Direct evaluation of the properties of the metrics using this analytic approach appears difficult because the metric formulas are too complicated. However, we can expand the formulas in power series of deviations of the random variates from their mean values. This leads to multinomial expressions in the random variates which are more easily evaluated.

Experimenting with this approach we find that a 4th order expansion appears adequate.

In[•]:=
```
order = 4;
```

Now we carry out this expansion, using ϵ as a bookkeeping parameter which we later set to 1 . We write SdEx for the expansion of Sd and OdEx for the expansion of Od (the resulting formulas are very large):

In[•]:=
```
SdEx = Normal[Series[Sd /. (# :→ EE[#] + ϵ (# - EE[#]) & /@ variables), {ϵ, 0, order}]] // Expand //
    PowerExpand // Expand;
OdEx = Normal[Series[Od /. (# :→ EE[#] + ϵ (# - EE[#]) & /@ variables), {ϵ, 0, order}]] // Expand //
    PowerExpand // Expand;
```

In this approximation we compute the expectation values of Sd (denoted ESdEx) and Od (EOSdEx) and also the expectation values of their squares Sd^2 (ESdEx2) and Od^2 (EOdEx2) to prepare computation of variances. In an intermediate steps below we are generating proper power series objects from SdEx and OdEx (called SdExx and OdExx) because these can be used to avoid computing terms of order higher than needed when taking squares.

In[•]:=
```
ESdEx = expectation /@ SdEx;
SdExx = Series[SdEx // Factor // PowerExpand // Expand, {ϵ, 0, order}];
ESdEx2 = expectation /@ Expand[Normal[SdExx ^ 2]];
```

In[•]:=
```
EOdEx = expectation /@ OdEx;
OdExx = Series[OdEx // Factor // PowerExpand // Expand, {ϵ, 0, order}];
EOdEx2 = expectation /@ Expand[Normal[OdExx ^ 2]];
```

After removing the bookkeeping parameter ϵ, this gives the variance of Od:

In[•]:=
```
varOd = Expand[EOdEx2 - Normal[Series[EOdEx, {ϵ, 0, order}] ^ 2]] /. ϵ → 1 // FullSimplify
```

Out[•]=
$$\frac{1}{32\, n^5\, \omega^2} (-1+\omega) \Big(16\, n^4\, \omega^2 (-4+3\,\omega) + 2\, n^3\, \omega^2 \big(-35 + n1 (32 - 48\,\omega) + 8\, n2 (2 - 5\,\omega) + 31\,\omega\big) + \omega \big(n2^2 (163 - 666\,\omega + 562\,\omega^2) + n1^2 (203 - 786\,\omega + 642\,\omega^2) + n1\, n2 (203 - 786\,\omega + 642\,\omega^2)\big) + n^2 \Big(-192 + \omega \big(715 + 2\,\omega (-385 + 62\, n1 + 35\, n2 + (121 + 24\, n1^2 + 3\, n2 (-21 + 8\, n2) + n1 (-86 + 24\, n2))\,\omega)\big)\Big) + n \Big(2\, n1^2\, \omega^2 (-27 + 55\,\omega) + n2 \big(96 + \omega (-555 + 2\,\omega (577 - 18\, n2 - 377\,\omega + 46\, n2\,\omega))\big) + 2\, n1 \big(96 + \omega (-459 + \omega (778 - 27\, n2 - 442\,\omega + 55\, n2\,\omega))\big)\Big)\Big)$$

And the variance of Sd :

In[•]:=
```
varSd = Expand[ESdEx2 - Normal[Series[ESdEx, {ϵ, 0, order}] ^ 2]] /. ϵ → 1 // FullSimplify
```

Out[•]=
$$\frac{1}{8\, n^5\, \omega^2} (-1+\omega) \Big(4\, n^4\, \omega^2 (-4+3\,\omega) + 2\, n^3\, \omega \big(4 + \omega (-19 + 8\, n1 + 4\, n2 + 13\,\omega - 2 (6\, n1 + 5\, n2)\,\omega)\big) + n^2 \Big(-48 + \omega \big(185 - 8\, n1 - 4\, n2 + 2 (-103 + 32\, n1 + 19\, n2)\,\omega + 2 (33 + 6\, n1^2 + n1 (-32 + 6\, n2) + n2 (-23 + 6\, n2))\,\omega^2\big)\Big) + \omega \big(9\, n2^2 (5 + 2\,\omega (-9 + 7\,\omega)) + n1^2 (65 + 2\,\omega (-111 + 83\,\omega)) + n1\, n2 (65 + 2\,\omega (-111 + 83\,\omega))\big) + n \Big(2\, n1^2\, \omega^2 (-13 + 19\,\omega) + n1 \big(48 + 2\,\omega (-125 + \omega (214 - 13\, n2 - 116\,\omega + 19\, n2\,\omega))\big) + n2 \big(24 + \omega (-149 + 2\,\omega (151 - 93\,\omega + 2\, n2 (-5 + 8\,\omega)))\big)\Big)\Big)$$

The following are the expectation values of Sd and Ed:

In[∘]:= ESdEx /. ϵ → 1 // FullSimplify

Out[∘]=
$$\frac{n2\left(-7+\omega\left(31-2\left(21+n+2\,n^2\right)\omega+2\left(9+n+2\,n^2+4\,n^3\right)\omega^2\right)\right)}{8\,n^4\,\omega^2}$$

In[∘]:= EOdEx /. ϵ → 1 // FullSimplify

Out[∘]=
$$\frac{n2\left(-29+\omega\left(131-6\,n+4\left(-45+n-4\,n^2\right)\omega+2\left(39+n+8\,n^2+32\,n^3\right)\omega^2\right)\right)}{64\,n^4\,\omega^2}$$

We test these results by comparison with direct simulations. (Readers are invited to perform further numerical tests. For some parameter combinations our 4th order approximation will be more, for others less accurate.)

In[∘]:=
```
num = {n → 50, n1 → 1, n2 → 10, ω → 0.8}; (* parameter choices *)
aSet = Table[i, {i, 1, n /. num}]; (* Indices of species in year a, etc. *)
bSet = Table[i + n1 /. nRules //. num, {i, 1, n /. num}];
cSet = Table[i + n1 + n2 /. nRules //. num, {i, 1, n /. num}];
differences = Table[(* Generate 100,000 replicates of observations *)
    aNObs = RandomVariate[BinomialDistribution[Length[aSet], ω /. num]];
    bNObs = RandomVariate[BinomialDistribution[Length[bSet], ω /. num]];
    cNObs = RandomVariate[BinomialDistribution[Length[cSet], ω /. num]];
    aObs = aSet[[RandomSample[aSet, aNObs]]];
    bObs = bSet[[RandomSample[aSet, bNObs]]];
    cObs = cSet[[RandomSample[aSet, cNObs]]];
    abNJoint = Length[Intersection[aObs, bObs]];
    acNJoint = Length[Intersection[aObs, cObs]];
    {abNJoint / Sqrt[aNObs * bNObs] - acNJoint / Sqrt[aNObs * cNObs],
      2 abNJoint / (aNObs + bNObs) - 2 acNJoint / (aNObs + cNObs)} // N,
    {100 000}];
```

In[∘]:=
```
Mean[differences] (* Simulated mean values of Od and Ed *)
{EOdEx, ESdEx} /. ϵ → 1 //. num (* Analytic prediction of means *)
Variance[differences] (* Simulated variances of Od and Ed *)
Differences[%] (* Simulated difference between variances *)
{varOd, varSd} //. num (* Analytic predictions of variances *)
Differences[%] (* Analytic difference between variances *)
```

Out[∘]= {0.160097, 0.159888}

Out[∘]= {0.159798, 0.159596}

Out[∘]= {0.00388987, 0.00390357}

Out[∘]= {0.0000136934}

Out[∘]= {0.00388092, 0.00389473}

Out[∘]= {0.0000138093}

The accuracy is better than 1%, for the difference between variances better than 5%.

Having confirmed the analytic approximations, we now ask whether there are parameter combinations for which the coefficient of variation (CV) of Od is larger than the CV of Sd or, equivalently, for which ESdEx^2*varOd - EOdEx^2*varSd > 0. So, the question is whether the following quantity can become positive for some valid parameter combination:

In[ ]:=
```
test = FullSimplify[ESdEx^2*varOd - EOdEx^2*varSd /. ϵ → 1]
```

Out[ ]=

$$\frac{1}{32768\, n^{13}\, \omega^6}\, n2^2 (-1+\omega) \Big(16 \big(7+\omega \big(-31+2 \omega \big(21+n+2 n^2-\big(9+n+2 n^2+4 n^3\big) \omega\big)\big)\big)^2$$
$$\big(16 n^4 \omega^2 (-4+3 \omega)+2 n^3 \omega^2 (-35+n1 (32-48 \omega)+8\, n2 (2-5 \omega)+31 \omega)+$$
$$\omega \big(n2^2 \big(163-666 \omega+562 \omega^2\big)+n1^2 \big(203-786 \omega+642 \omega^2\big)+n1\, n2 \big(203-786 \omega+642 \omega^2\big)\big)+$$
$$n^2 \big(-192+\omega \big(715+2 \omega \big(-385+62\, n1+35\, n2+\big(121+24\, n1^2+3\, n2 (-21+8\, n2)+n1 (-86+24\, n2)\big) \omega\big)\big)\big)+$$
$$n \big(2\, n1^2 \omega^2 (-27+55 \omega)+n2 (96+\omega (-555+2 \omega (577-18\, n2-377 \omega+46\, n2\, \omega)))+$$
$$2\, n1 (96+\omega (-459+\omega (778-27\, n2-442 \omega+55\, n2\, \omega))))\big)-$$
$$\big(-29+\omega \big(131-6 n+4 \big(-45+n-4 n^2\big) \omega+2 \big(39+n+8 n^2+32 n^3\big) \omega^2\big)\big)^2$$
$$\big(4 n^4 \omega^2 (-4+3 \omega)+2 n^3 \omega (4+\omega (-19+8\, n1+4\, n2+13 \omega-2 (6\, n1+5\, n2) \omega))+n^2 \big(-48+\omega \big(185-8\, n1-$$
$$4\, n2+2 (-103+32\, n1+19\, n2) \omega+2 \big(33+6\, n1^2+n1 (-32+6\, n2)+n2 (-23+6\, n2)\big) \omega^2\big)\big)+$$
$$\omega \big(9\, n2^2 (5+2 \omega (-9+7 \omega))+n1^2 (65+2 \omega (-111+83 \omega))+n1\, n2 (65+2 \omega (-111+83 \omega))\big)+$$
$$n \big(2\, n1^2 \omega^2 (-13+19 \omega)+n1 (48+2 \omega (-125+\omega (214-13\, n2-116 \omega+19\, n2\, \omega)))+$$
$$n2 (24+\omega (-149+2 \omega (151-93 \omega+2\, n2 (-5+8 \omega))))\big)\big)\Big)$$

To answer this question we use the FindInstance function of Mathematica, which is guaranteed to return such parameter combinations if they exist upon termination. Unfortunately, this call of FindInstance does not appear to terminate end over several hours.

In[ ]:=
```
(* The following does not stop in any reasonable time:*)
(*fi=FindInstance[
   $Assumptions &&n>(n1+n2)&&3n/4>n1≥1&&n2≥1&&1/3≤ω<1&&Element[n,Integers]&&Element[n1,
     Integers]&&Element[n2,Integers]&&test>0//Evaluate,{n,n2,n1,ω}]//AbsoluteTiming*)
```

To simplify the computational problem, we therefore fix the value of $\omega$ and ask whether there are values of n, n1, and n2 such that the CV of Od becomes larger than the CV of Sd. This computation terminates much faster. We vary $\omega$ in steps of 0.01 from 0.01 to 0.99.

We restrict n1 to be less than 3/4 of n (that is, less than 3/4 of species are new between years a and b, which is a plausible assumption). We are doing this because it generates simpler results.

The output of this test, executed below, has the format:

```
{<ω>, <computation time>,
 < set of one or zero example parameter combinations for which CV of Od is larger than
CV of Sd}
```

```
In[◦]:= resultsByOmega = Table[
    {omega100 / 100, fi = FindInstance[$Assumptions && n > (n1 + n2) && 3 n / 4 > n1 ≥ 1 && n2 ≥ 1 &&
            Element[n, Integers] && Element[n1, Integers] && Element[n2, Integers] && test > 0 /.
          ω → omega100 / 100 // Evaluate, {n1, n2, n}] // AbsoluteTiming;
     Print[Prepend[fi, omega100 / 100 // N]]; fi},
    {omega100, 1, 99}];

(* Generate graphic illustrating the result: *)
ListPlot[{resultsByOmega[[ ;; , 1]], 1 - Length /@ resultsByOmega[[ ;; , 2, 2]]} // Transpose,
 Filling → Axis, Frame → {{True, False}, {True, False}},
 FrameLabel → {{"Ochiai always better", None}, {"Observation probability ω", None}},
 FrameTicks → {{{{1, "Yes"}, {0, "No"}}, None}, {Automatic, None}}]

{0.01, 1.93432, {{n1 → 1, n2 → 1, n → 3}}}
{0.02, 1.70041, {{n1 → 1, n2 → 1, n → 3}}}
{0.03, 1.9381, {{n1 → 1, n2 → 1, n → 3}}}
{0.04, 1.46324, {{n1 → 1, n2 → 1, n → 3}}}
{0.05, 1.84105, {{n1 → 1, n2 → 1, n → 3}}}
{0.06, 1.97892, {{n1 → 1, n2 → 1, n → 3}}}
{0.07, 2.10242, {{n1 → 1, n2 → 1, n → 3}}}
{0.08, 1.65497, {{n1 → 1, n2 → 1, n → 3}}}
{0.09, 2.09676, {{n1 → 1, n2 → 1, n → 3}}}
{0.1, 2.31601, {{n1 → 1, n2 → 1, n → 3}}}
{0.11, 2.20776, {{n1 → 1, n2 → 1, n → 3}}}
{0.12, 1.62323, {{n1 → 1, n2 → 1, n → 3}}}
{0.13, 2.61085, {{n1 → 1, n2 → 1, n → 3}}}
{0.14, 2.27466, {{n1 → 1, n2 → 1, n → 3}}}
{0.15, 1.79229, {{n1 → 1, n2 → 1, n → 3}}}
{0.16, 1.99882, {{n1 → 1, n2 → 1, n → 3}}}
{0.17, 2.47031, {{n1 → 1, n2 → 1, n → 3}}}
{0.18, 2.19295, {{n1 → 1, n2 → 1, n → 3}}}
{0.19, 2.59631, {{n1 → 1, n2 → 1, n → 3}}}
{0.2, 2.50319, {{n1 → 1, n2 → 1, n → 3}}}
{0.21, 3.15013, {{n1 → 1, n2 → 1, n → 3}}}
{0.22, 4.32749, {{n1 → 1, n2 → 1, n → 3}}}
{0.23, 3.53445, {{n1 → 1, n2 → 1, n → 3}}}
{0.24, 4.3355, {}}
{0.25, 3.11287, {}}
```

{0.26, 4.79726, {}}

{0.27, 4.18818, {}}

{0.28, 3.42542, {}}

{0.29, 4.52998, {}}

{0.3, 2.99154, {}}

{0.31, 4.67204, {}}

{0.32, 3.87643, {}}

{0.33, 5.22752, {}}

{0.34, 5.16305, {}}

{0.35, 2.92864, {}}

{0.36, 3.33823, {}}

{0.37, 3.27086, {}}

{0.38, 2.87953, {}}

{0.39, 3.40394, {}}

{0.4, 2.00197, {}}

{0.41, 2.61061, {}}

{0.42, 2.24467, {}}

{0.43, 2.81331, {}}

{0.44, 2.07886, {}}

{0.45, 1.90414, {}}

{0.46, 2.41863, {}}

{0.47, 2.60495, {}}

{0.48, 1.85915, {}}

{0.49, 3.25576, {}}

{0.5, 0.816236, {}}

{0.51, 2.20724, {}}

{0.52, 1.99844, {}}

{0.53, 2.39279, {}}

{0.54, 1.99135, {}}

{0.55, 2.02243, {}}

{0.56, 1.94371, {}}

{0.57, 2.63322, {}}

{0.58, 2.28716, {}}

{0.59, 2.61369, {}}

{0.6, 1.15788, {}}

{0.61, 2.60824, {}}

{0.62, 2.23974, {}}
{0.63, 2.45199, {}}
{0.64, 2.06425, {}}
{0.65, 1.88982, {}}
{0.66, 2.5594, {}}
{0.67, 3.15126, {}}
{0.68, 2.03455, {}}
{0.69, 2.65277, {}}
{0.7, 1.53894, {}}
{0.71, 2.56374, {}}
{0.72, 1.81853, {}}
{0.73, 2.55395, {}}
{0.74, 2.2205, {}}
{0.75, 1.16938, {}}
{0.76, 1.98524, {}}
{0.77, 2.52931, {}}
{0.78, 2.11108, {}}
{0.79, 2.49302, {}}
{0.8, 1.18458, {}}
{0.81, 2.25273, {}}
{0.82, 2.37479, {}}
{0.83, 2.45286, {}}
{0.84, 1.73377, {}}
{0.85, 1.67768, {}}
{0.86, 2.16009, {}}
{0.87, 2.45632, {}}
{0.88, 2.05322, {}}
{0.89, 2.64172, {}}
{0.9, 1.45678, {}}
{0.91, 2.55837, {}}
{0.92, 1.90688, {}}
{0.93, 2.55987, {}}
{0.94, 2.41667, {}}
{0.95, 2.17444, {}}
{0.96, 2.23538, {}}
{0.97, 3.32419, {}}

```
{0.98, 2.84096, {}}
{0.99, 3.26571, {}}
```

Out[•]=

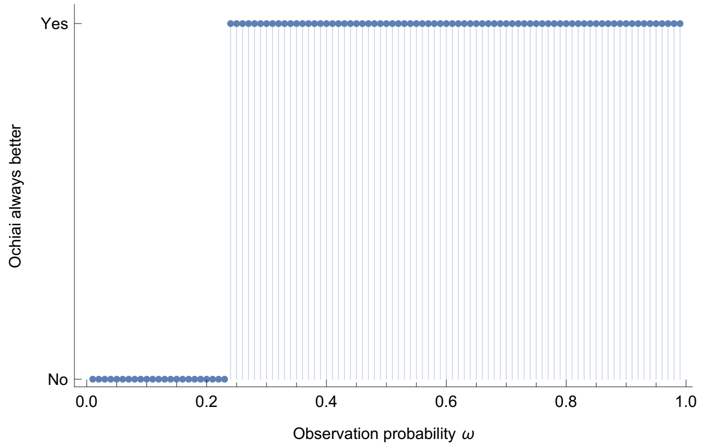


From this output we conclude that for n1 < 3 n/4 (less then 3/4 of species are new in year b) and at least when $\omega > 1/3$ (i.e., more than 1/3 of present species are typically observed) the CV of Od is always smaller than the CV of Sd, irrespective of the specific values of $\omega$, n, n1 and n2.

That is, the difference between years b and c in their similarity with year a is more reproducibly determined using Ochiai than Sørensen similarity. Our determinations of turnover rate from such differences using Ochiai similarity can on this basis be expected to be more accurate than corresponding determinations using Sørensen similarity would be.
