# Supplementary material for: Widespread slowdown in short-term species turnover despite accelerating climate change
Source: Nat Commun. 2026 Feb 3;17:1450. doi: 10.1038/s41467-025-68187-1 (PMC12886849; doi:10.1038/s41467-025-68187-1)
Supplement: Supplementary file 2 — Description of Additional Supplementary Files [file 41467_2025_68187_MOESM2_ESM.pdf]

Description of Additional Supplementary Files

Supplementary Code 1: Method Comparison

A numerical comparison of the accuracy of two methods to determine turnover rate from the slope of decline in similarity, which demonstrates that, when directed, extrinsically driven species turnover dominates even over short lags, our method does not sacrifice accuracy compared to a method comparing communities over lag periods as long as the entire time series.
