# Supplementary material for: Widespread slowdown in short-term species turnover despite accelerating climate change
Source: Nat Commun. 2026 Feb 3;17:1450. doi: 10.1038/s41467-025-68187-1 (PMC12886849; doi:10.1038/s41467-025-68187-1)
Supplement: Supplementary file 4 — Reporting Summary [file 41467_2025_68187_MOESM4_ESM.pdf]

nature portfolio

Corresponding author(s): Axel G. Rossberg

Last updated by author(s): Sep 19, 2025

Reporting Summary

Nature Portfolio wishes to improve the reproducibility of the work that we publish. This form provides structure for consistency and transparency in reporting. For further information on Nature Portfolio policies, see our Editorial Policies and the Editorial Policy Checklist.

Statistics

For all statistical analyses, confirm that the following items are present in the figure legend, table legend, main text, or Methods section.

n/a	Confirmed	
☐	☒	The exact sample size (*n*) for each experimental group/condition, given as a discrete number and unit of measurement
☐	☒	A statement on whether measurements were taken from distinct samples or whether the same sample was measured repeatedly
☐	☒	The statistical test(s) used AND whether they are one- or two-sided<br>*Only common tests should be described solely by name; describe more complex techniques in the Methods section.*
☐	☒	A description of all covariates tested
☐	☒	A description of any assumptions or corrections, such as tests of normality and adjustment for multiple comparisons
☐	☒	A full description of the statistical parameters including central tendency (e.g. means) or other basic estimates (e.g. regression coefficient) AND variation (e.g. standard deviation) or associated estimates of uncertainty (e.g. confidence intervals)
☐	☒	For null hypothesis testing, the test statistic (e.g. *F*, *t*, *r*) with confidence intervals, effect sizes, degrees of freedom and *P* value noted<br>*Give P values as exact values whenever suitable.*
☒	☐	For Bayesian analysis, information on the choice of priors and Markov chain Monte Carlo settings
☒	☐	For hierarchical and complex designs, identification of the appropriate level for tests and full reporting of outcomes
☒	☐	Estimates of effect sizes (e.g. Cohen's *d*, Pearson's *r*), indicating how they were calculated

Our web collection on statistics for biologists contains articles on many of the points above.

Software and code

Policy information about availability of computer code

Data collection	The data used for this study was sourced from the BioTIME database. These studies involved recording the presence of species in different locations with no software used in data collection study.
Data analysis	The data analyses in this study were conducted using R version 4.4.1. The code is available from https://zenodo.org/records/17791136.

For manuscripts utilizing custom algorithms or software that are central to the research but not yet described in published literature, software must be made available to editors and reviewers. We strongly encourage code deposition in a community repository (e.g. GitHub). See the Nature Portfolio guidelines for submitting code & software for further information.

Data

Policy information about availability of data

All manuscripts must include a data availability statement. This statement should provide the following information, where applicable:

- Accession codes, unique identifiers, or web links for publicly available datasets
- A description of any restrictions on data availability
- For clinical datasets or third party data, please ensure that the statement adheres to our policy

The data that support the findings of this study were sourced from the BioTIME database. Intermediate results from the analysis are provided at https://zenodo.org/records/17791136.

Research involving human participants, their data, or biological material

Policy information about studies with human participants or human data. See also policy information about sex, gender (identity/presentation), and sexual orientation and race, ethnicity and racism.

Reporting on sex and gender	No sex- and gender-based analyses have been performed because the study is a non-human study involving only observing species' presence or absence in their natural habitats.
Reporting on race, ethnicity, or other socially relevant groupings	The study did not involve human participants and required no socially constructed or socially relevant categorization variables in our manuscript.
Population characteristics	Our study is non-human; hence, no relevant population characteristics of human participants were included in the manuscript.
Recruitment	No human participant recruitment was involved in our study.
Ethics oversight	Because no human was involved in our study, no relevant approval from any organization was required.

Note that full information on the approval of the study protocol must also be provided in the manuscript.

Field-specific reporting

Please select the one below that is the best fit for your research. If you are not sure, read the appropriate sections before making your selection.

☐ Life sciences ☐ Behavioural & social sciences ☒ Ecological, evolutionary & environmental sciences

For a reference copy of the document with all sections, see nature.com/documents/nr-reporting-summary-flat.pdf

Life sciences study design

All studies must disclose on these points even when the disclosure is negative.

Sample size	*Describe how sample size was determined, detailing any statistical methods used to predetermine sample size OR if no sample-size calculation was performed, describe how sample sizes were chosen and provide a rationale for why these sample sizes are sufficient.*
Data exclusions	*Describe any data exclusions. If no data were excluded from the analyses, state so OR if data were excluded, describe the exclusions and the rationale behind them, indicating whether exclusion criteria were pre-established.*
Replication	*Describe the measures taken to verify the reproducibility of the experimental findings. If all attempts at replication were successful, confirm this OR if there are any findings that were not replicated or cannot be reproduced, note this and describe why.*
Randomization	*Describe how samples/organisms/participants were allocated into experimental groups. If allocation was not random, describe how covariates were controlled OR if this is not relevant to your study, explain why.*
Blinding	*Describe whether the investigators were blinded to group allocation during data collection and/or analysis. If blinding was not possible, describe why OR explain why blinding was not relevant to your study.*

Behavioural & social sciences study design

All studies must disclose on these points even when the disclosure is negative.

Study description	*Briefly describe the study type including whether data are quantitative, qualitative, or mixed-methods (e.g. qualitative cross-sectional, quantitative experimental, mixed-methods case study).*
Research sample	*State the research sample (e.g. Harvard university undergraduates, villagers in rural India) and provide relevant demographic information (e.g. age, sex) and indicate whether the sample is representative. Provide a rationale for the study sample chosen. For studies involving existing datasets, please describe the dataset and source.*
Sampling strategy	*Describe the sampling procedure (e.g. random, snowball, stratified, convenience). Describe the statistical methods that were used to predetermine sample size OR if no sample-size calculation was performed, describe how sample sizes were chosen and provide a rationale for why these sample sizes are sufficient. For qualitative data, please indicate whether data saturation was considered, and what criteria were used to decide that no further sampling was needed.*
Data collection	*Provide details about the data collection procedure, including the instruments or devices used to record the data (e.g. pen and paper, computer, eye tracker, video or audio equipment) whether anyone was present besides the participant(s) and the researcher, and whether the researcher was blind to experimental condition and/or the study hypothesis during data collection.*

Timing

Indicate the start and stop dates of data collection. If there is a gap between collection periods, state the dates for each sample cohort.

Data exclusions

If no data were excluded from the analyses, state so OR if data were excluded, provide the exact number of exclusions and the rationale behind them, indicating whether exclusion criteria were pre-established.

Non-participation

State how many participants dropped out/declined participation and the reason(s) given OR provide response rate OR state that no participants dropped out/declined participation.

Randomization

If participants were not allocated into experimental groups, state so OR describe how participants were allocated to groups, and if allocation was not random, describe how covariates were controlled.

Ecological, evolutionary & environmental sciences study design

All studies must disclose on these points even when the disclosure is negative.

Study description

Our study investigates the drivers of temporal species turnover, focusing on whether environmental changes or intrinsic ecosystem dynamics are the primary factors. Using the BioTIME database, we analyzed long-term changes in species composition across various ecosystems over the past 100 years. The study expected either an acceleration of turnover with accelerating climate change or constant turnover if intrinsic mechanisms dominate. Surprisingly, it found that species turnover has decelerated in significantly more ecosystems, with turnover rates typically declining by one-third.
Quantitative Data:
Treatment Factors and Interactions: The study considered environmental shifts (e.g., temperature, precipitation) and intrinsic ecosystem dynamics (e.g., ecological drift, species interactions).
Design Structure: The study analyzed community time series data partitioned into local communities.
Nature and Number of Experimental Units: The study included 4,863,824 records of species observations from 189 studies, covering 15,469 species across seven guilds (plants, fish, birds, mammals, reptiles, invertebrates, benthic and mixed communities) from freshwater, marine, and terrestrial ecosystems.
Replicates: The study used multiple time series from different ecosystems.
In our study, we developed a novel methodology to assess turnover rates, comparing periods before and after the acceleration of environmental shifts around 1975. We used the Ochiai metric for community similarity and robust statistical methods to ensure the reliability of the results.

Research sample

Research Sample:
The research sample consists of extensive community composition time series data from the BioTIME database. This database includes 4,863,824 records of species observations from 189 studies, covering 15,469 species across various taxa and ecosystems.

Organism Taxa:
Taxa: The study includes plants, fish, birds, mammals, reptiles, invertebrates, benthic organisms, and mixed communities.
Source: The data were sourced from the BioTIME database, which compiles biodiversity time series from various published and unpublished studies.
Sex and Age Range: The data source did not specify sex or age range as it focuses on community-level data rather than individual organisms.

Rationale for Sample Choice:
The BioTIME database was chosen because it provides a comprehensive and well-documented collection of biodiversity time series data. This allows for robust analysis of long-term trends in species turnover across different ecosystems and taxa. This extensive dataset enables the study to draw more generalizable conclusions about the drivers of species turnover. We used the BioTIME database as published to avoid confirmation bias.

Population Representation:
The sample is meant to represent ecological communities from a wide range of ecosystems, including freshwater, marine, and terrestrial habitats. The study aims to infer general patterns and drivers of species turnover that could apply to similar ecosystems globally.

Data and Source:
Data: The study analyzed community composition datasets with considerable temporal extent (up to 44 years) and spatial coverage.
Source: The BioTIME database includes data from various studies on biodiversity time series.

Manipulations:
Our study did not involve direct manipulations of the organisms or ecosystems. Instead, we analyzed existing datasets to assess changes in species turnover rates over time.
Our study leverages the BioTIME database, a rich source of long-term ecological data, to investigate the relative contributions of environmental changes and intrinsic ecosystem dynamics to species turnover.

Sampling strategy

It was irrelevant for us to perform a predetermined sample size calculation, considering we sourced our data from an existing database. The sample sizes were determined by data availability in the BioTIME database. The study included 4,863,824 records of species observations from 189 studies, covering 15,469 species.

The large and diverse dataset from the BioTIME database provides a robust basis for analyzing long-term trends in species turnover. The database's extensive temporal and spatial coverage ensures that the findings are generalizable across different ecosystems and taxa.

Data collection	The BioTIME database documentation provides details of the data collection procedure, including who recorded the data and how.
Timing and spatial scale	Data Collection Period: The earliest data in the BioTIME database used dates back to 1927, and the most recent data extends to 2015.<br><br>Frequency and Periodicity of Sampling:<br>The frequency of sampling varied across different studies within the BioTIME database. Some studies had annual sampling, while others had different periodicities. Our analysis considered annual community compositions.<br><br>Rationale for Sampling Frequency and Periodicity:<br>The varied frequency and periodicity of sampling reflect the diverse nature of the studies included in the BioTIME database. This diversity allows for a comprehensive analysis of species turnover across different ecosystems and time scales.<br><br>Gaps Between Collection Periods:<br>In some community time series, we observed gaps in the survey data. In such cases, we maintained the temporal survey period by replacing the missing years with NAs. We included all community time series that started more than 5 years before and ended at least 5 years after the chosen breakpoint years.<br><br>Spatial Scale:<br>Spatial Coverage: The data were collected from a wide range of ecosystems, including freshwater, marine, and terrestrial habitats.<br>Spatial Partitioning: Studies covering more than 96 km^2 were partitioned into separate local communities to ensure that the analysis was conducted at a relevant spatial scale.<br><br>Rationale for Spatial Scale:<br>The broad spatial coverage and partitioning protocol ensure that the study captures a wide range of ecological dynamics and provides a robust basis for analyzing species turnover across different ecosystems. By leveraging the extensive temporal and spatial coverage of the BioTIME database, we provide a comprehensive analysis of species turnover, accounting for intrinsic ecosystem dynamics and environmental changes.
Data exclusions	Data Exclusions:<br>Some data were excluded from the analyses. The exclusions and their rationale are as follows:<br>Early Survey Data:<br>Exclusion: Data from survey dates earlier than 1927 were filtered out.<br>Rationale: Early survey techniques, skills, and interests were evolving, which may have introduced large differences and potential biases in the data. Excluding these early data helps ensure consistency and reliability in the analysis.<br>Incomplete Time Series:<br>Exclusion: Community time series that did not start more than 5 years before and end at least 5 years after the chosen breakpoint years were excluded.<br>Rationale: Ensuring that each time series spans a sufficient period before and after the breakpoint years is crucial for accurately assessing changes in turnover rates.<br><br>Insufficient Data Points for Regression:<br>Exclusion: Communities with less than three data points available for the regression of mean similarity against lag were excluded.<br>Rationale: A minimum number of data points is necessary to perform a reliable regression analysis and compute turnover rates accurately.<br><br>Pre-Established Exclusion Criteria:<br>The exclusion criteria were pre-established to ensure the analysis's robustness and reliability. These criteria were designed to minimize biases and ensure that the data used in the study were suitable for the intended analyses.<br><br>Further Exclusion: community/breakyear combinations where the estimated turnover rate before the breakpoint equaled exactly the estimated rate since (usually both being zero) were excluded.<br>Rationale: for these communities the number of sampled species was usually small. The nominal results of no change in turnover rate is therefore likely to be biased by low taxonomic coverage. In any case, zeros are commonly excluded from the sign test that we perform.
Reproducibility	Measures to verify reproducibility:<br>Robustness analysis:<br>Different metrics: The analysis was repeated using various metrics of community similarity (e.g., Jaccard, Sørensen) to ensure that the findings were not dependent on a specific metric.<br><br>Longer lags: To verify the consistency of the results, the analysis was also repeated, considering community changes over lags longer than five years.<br><br>Exclusion of specific data:<br>Sample completeness: The analysis was repeated after omitting a step filtering for 'sample completeness' to check the impact of this step on the results.<br><br>Treatment studies: The analysis was repeated after removing all studies for which BioTIME registers a 'treatment' to ensure that intentional manipulations of study communities did not influence the results.<br><br>Separate analyses for community types:<br>The analysis was conducted separately for the main community types (benthos, fish, birds, and mixed communities) to verify the generality of the observed patterns across different community types.

	Results of reproducibility attempts:<br>Successful repetition: All attempts to repeat the experiments were successful. The patterns observed in the overall results were consistent across different metrics, longer lags, and after excluding specific data subsets.<br><br>Consistency across community types: The observed slowdown in species turnover was replicated independently for mixed, bird, and benthic communities. There was no consistent signal for fish communities, likely due to the influence of fisheries management.<br><br>Our measures to verify reproducibility demonstrated that the findings were robust and consistent across various conditions and community types.
Randomization	In this study, allocation of samples into groups was not relevant because our analysis was based on existing time series data from the BioTIME database. Our study did not involve experimental manipulation or the creation of treatment and control groups. Instead, we analyzed observational data collected over time from various ecological communities.
Blinding	Blinding was irrelevant in our study because we analyzed existing time series data from the BioTIME database.

Did the study involve field work? ☐ Yes ☒ No

Field work, collection and transport

Field conditions	*Describe the study conditions for field work, providing relevant parameters (e.g. temperature, rainfall).*
Location	*State the location of the sampling or experiment, providing relevant parameters (e.g. latitude and longitude, elevation, water depth).*
Access & import/export	*Describe the efforts you have made to access habitats and to collect and import/export your samples in a responsible manner and in compliance with local, national and international laws, noting any permits that were obtained (give the name of the issuing authority, the date of issue, and any identifying information).*
Disturbance	*Describe any disturbance caused by the study and how it was minimized.*

Reporting for specific materials, systems and methods

We require information from authors about some types of materials, experimental systems and methods used in many studies. Here, indicate whether each material, system or method listed is relevant to your study. If you are not sure if a list item applies to your research, read the appropriate section before selecting a response.

Materials & experimental systems

n/a	Involved in the study
☒	☐ Antibodies
☒	☐ Eukaryotic cell lines
☒	☐ Palaeontology and archaeology
☒	☐ Animals and other organisms
☒	☐ Clinical data
☒	☐ Dual use research of concern
☒	☐ Plants

Methods

n/a	Involved in the study
☒	☐ ChIP-seq
☒	☐ Flow cytometry
☒	☐ MRI-based neuroimaging

Antibodies

Antibodies used	*Describe all antibodies used in the study; as applicable, provide supplier name, catalog number, clone name, and lot number.*
Validation	*Describe the validation of each primary antibody for the species and application, noting any validation statements on the manufacturer's website, relevant citations, antibody profiles in online databases, or data provided in the manuscript.*

Eukaryotic cell lines

Policy information about cell lines and Sex and Gender in Research

Cell line source(s)	*State the source of each cell line used and the sex of all primary cell lines and cells derived from human participants or vertebrate models.*
Authentication	*Describe the authentication procedures for each cell line used OR declare that none of the cell lines used were authenticated.*
Mycoplasma contamination	*Confirm that all cell lines tested negative for mycoplasma contamination OR describe the results of the testing for*

Mycoplasma contamination	*mycoplasma contamination OR declare that the cell lines were not tested for mycoplasma contamination.*
Commonly misidentified lines (See ICLAC register)	*Name any commonly misidentified cell lines used in the study and provide a rationale for their use.*

Palaeontology and Archaeology

Specimen provenance	*Provide provenance information for specimens and describe permits that were obtained for the work (including the name of the issuing authority, the date of issue, and any identifying information). Permits should encompass collection and, where applicable, export.*
Specimen deposition	*Indicate where the specimens have been deposited to permit free access by other researchers.*
Dating methods	*If new dates are provided, describe how they were obtained (e.g. collection, storage, sample pretreatment and measurement), where they were obtained (i.e. lab name), the calibration program and the protocol for quality assurance OR state that no new dates are provided.*

☐ Tick this box to confirm that the raw and calibrated dates are available in the paper or in Supplementary Information.

Ethics oversight	*Identify the organization(s) that approved or provided guidance on the study protocol, OR state that no ethical approval or guidance was required and explain why not.*

Note that full information on the approval of the study protocol must also be provided in the manuscript.

Animals and other research organisms

Policy information about studies involving animals; ARRIVE guidelines recommended for reporting animal research, and Sex and Gender in Research

Laboratory animals	*For laboratory animals, report species, strain and age OR state that the study did not involve laboratory animals.*
Wild animals	*Provide details on animals observed in or captured in the field; report species and age where possible. Describe how animals were caught and transported and what happened to captive animals after the study (if killed, explain why and describe method; if released, say where and when) OR state that the study did not involve wild animals.*
Reporting on sex	*Indicate if findings apply to only one sex; describe whether sex was considered in study design, methods used for assigning sex. Provide data disaggregated for sex where this information has been collected in the source data as appropriate; provide overall numbers in this Reporting Summary. Please state if this information has not been collected. Report sex-based analyses where performed, justify reasons for lack of sex-based analysis.*
Field-collected samples	*For laboratory work with field-collected samples, describe all relevant parameters such as housing, maintenance, temperature, photoperiod and end-of-experiment protocol OR state that the study did not involve samples collected from the field.*
Ethics oversight	*Identify the organization(s) that approved or provided guidance on the study protocol, OR state that no ethical approval or guidance was required and explain why not.*

Note that full information on the approval of the study protocol must also be provided in the manuscript.

Clinical data

Policy information about clinical studies

All manuscripts should comply with the ICMJE guidelines for publication of clinical research and a completed CONSORT checklist must be included with all submissions.

Clinical trial registration	*Provide the trial registration number from ClinicalTrials.gov or an equivalent agency.*
Study protocol	*Note where the full trial protocol can be accessed OR if not available, explain why.*
Data collection	*Describe the settings and locales of data collection, noting the time periods of recruitment and data collection.*
Outcomes	*Describe how you pre-defined primary and secondary outcome measures and how you assessed these measures.*

Dual use research of concern

Policy information about dual use research of concern

Hazards

Could the accidental, deliberate or reckless misuse of agents or technologies generated in the work, or the application of information presented in the manuscript, pose a threat to:

No	Yes	
☒	☐	Public health
☒	☐	National security
☒	☐	Crops and/or livestock
☒	☐	Ecosystems
☒	☐	Any other significant area

Experiments of concern

Does the work involve any of these experiments of concern:

No	Yes	
☒	☐	Demonstrate how to render a vaccine ineffective
☒	☐	Confer resistance to therapeutically useful antibiotics or antiviral agents
☒	☐	Enhance the virulence of a pathogen or render a nonpathogen virulent
☒	☐	Increase transmissibility of a pathogen
☒	☐	Alter the host range of a pathogen
☒	☐	Enable evasion of diagnostic/detection modalities
☒	☐	Enable the weaponization of a biological agent or toxin
☒	☐	Any other potentially harmful combination of experiments and agents

Plants

Seed stocks

Report on the source of all seed stocks or other plant material used. If applicable, state the seed stock centre and catalogue number. If plant specimens were collected from the field, describe the collection location, date and sampling procedures.

Novel plant genotypes

Describe the methods by which all novel plant genotypes were produced. This includes those generated by transgenic approaches, gene editing, chemical/radiation-based mutagenesis and hybridization. For transgenic lines, describe the transformation method, the number of independent lines analyzed and the generation upon which experiments were performed. For gene-edited lines, describe the editor used, the endogenous sequence targeted for editing, the targeting guide RNA sequence (if applicable) and how the editor was applied.

Authentication

Describe any authentication procedures for each seed stock used or novel genotype generated. Describe any experiments used to assess the effect of a mutation and, where applicable, how potential secondary effects (e.g. second site T-DNA insertions, mosiacism, off-target gene editing) were examined.

ChIP-seq

Data deposition

☐ Confirm that both raw and final processed data have been deposited in a public database such as GEO.

☐ Confirm that you have deposited or provided access to graph files (e.g. BED files) for the called peaks.

Data access links
May remain private before publication.

For "Initial submission" or "Revised version" documents, provide reviewer access links. For your "Final submission" document, provide a link to the deposited data.

Files in database submission

Provide a list of all files available in the database submission.

Genome browser session
(e.g. UCSC)

Provide a link to an anonymized genome browser session for "Initial submission" and "Revised version" documents only, to enable peer review. Write "no longer applicable" for "Final submission" documents.

Methodology

Replicates

Describe the experimental replicates, specifying number, type and replicate agreement.

Sequencing depth

Describe the sequencing depth for each experiment, providing the total number of reads, uniquely mapped reads, length of reads and whether they were paired- or single-end.

Antibodies

Describe the antibodies used for the ChIP-seq experiments; as applicable, provide supplier name, catalog number, clone name, and lot number.

Peak calling parameters	*Specify the command line program and parameters used for read mapping and peak calling, including the ChIP, control and index files used.*
Data quality	*Describe the methods used to ensure data quality in full detail, including how many peaks are at FDR 5% and above 5-fold enrichment.*
Software	*Describe the software used to collect and analyze the ChIP-seq data. For custom code that has been deposited into a community repository, provide accession details.*

Flow Cytometry

Plots

Confirm that:

☐ The axis labels state the marker and fluorochrome used (e.g. CD4-FITC).

☐ The axis scales are clearly visible. Include numbers along axes only for bottom left plot of group (a 'group' is an analysis of identical markers).

☐ All plots are contour plots with outliers or pseudocolor plots.

☐ A numerical value for number of cells or percentage (with statistics) is provided.

Methodology

Sample preparation	*Describe the sample preparation, detailing the biological source of the cells and any tissue processing steps used.*
Instrument	*Identify the instrument used for data collection, specifying make and model number.*
Software	*Describe the software used to collect and analyze the flow cytometry data. For custom code that has been deposited into a community repository, provide accession details.*
Cell population abundance	*Describe the abundance of the relevant cell populations within post-sort fractions, providing details on the purity of the samples and how it was determined.*
Gating strategy	*Describe the gating strategy used for all relevant experiments, specifying the preliminary FSC/SSC gates of the starting cell population, indicating where boundaries between "positive" and "negative" staining cell populations are defined.*

☐ Tick this box to confirm that a figure exemplifying the gating strategy is provided in the Supplementary Information.

Magnetic resonance imaging

Experimental design

Design type	*Indicate task or resting state; event-related or block design.*
Design specifications	*Specify the number of blocks, trials or experimental units per session and/or subject, and specify the length of each trial or block (if trials are blocked) and interval between trials.*
Behavioral performance measures	*State number and/or type of variables recorded (e.g. correct button press, response time) and what statistics were used to establish that the subjects were performing the task as expected (e.g. mean, range, and/or standard deviation across subjects).*

Acquisition

Imaging type(s)	*Specify: functional, structural, diffusion, perfusion.*
Field strength	*Specify in Tesla*
Sequence & imaging parameters	*Specify the pulse sequence type (gradient echo, spin echo, etc.), imaging type (EPI, spiral, etc.), field of view, matrix size, slice thickness, orientation and TE/TR/flip angle.*
Area of acquisition	*State whether a whole brain scan was used OR define the area of acquisition, describing how the region was determined.*

Diffusion MRI ☐ Used ☐ Not used

Preprocessing

Preprocessing software	*Provide detail on software version and revision number and on specific parameters (model/functions, brain extraction, segmentation, smoothing kernel size, etc.).*

Normalization	*If data were normalized/standardized, describe the approach(es): specify linear or non-linear and define image types used for transformation OR indicate that data were not normalized and explain rationale for lack of normalization.*
Normalization template	*Describe the template used for normalization/transformation, specifying subject space or group standardized space (e.g. original Talairach, MNI305, ICBM152) OR indicate that the data were not normalized.*
Noise and artifact removal	*Describe your procedure(s) for artifact and structured noise removal, specifying motion parameters, tissue signals and physiological signals (heart rate, respiration).*
Volume censoring	*Define your software and/or method and criteria for volume censoring, and state the extent of such censoring.*

Statistical modeling & inference

Model type and settings	*Specify type (mass univariate, multivariate, RSA, predictive, etc.) and describe essential details of the model at the first and second levels (e.g. fixed, random or mixed effects; drift or auto-correlation).*
Effect(s) tested	*Define precise effect in terms of the task or stimulus conditions instead of psychological concepts and indicate whether ANOVA or factorial designs were used.*

Specify type of analysis: ☐ Whole brain ☐ ROI-based ☐ Both

Statistic type for inference<br>(See Eklund et al. 2016)	*Specify voxel-wise or cluster-wise and report all relevant parameters for cluster-wise methods.*
Correction	*Describe the type of correction and how it is obtained for multiple comparisons (e.g. FWE, FDR, permutation or Monte Carlo).*

Models & analysis

n/a	Involved in the study
☐	☐ Functional and/or effective connectivity
☐	☐ Graph analysis
☐	☐ Multivariate modeling or predictive analysis

Functional and/or effective connectivity	*Report the measures of dependence used and the model details (e.g. Pearson correlation, partial correlation, mutual information).*
Graph analysis	*Report the dependent variable and connectivity measure, specifying weighted graph or binarized graph, subject- or group-level, and the global and/or node summaries used (e.g. clustering coefficient, efficiency, etc.).*
Multivariate modeling and predictive analysis	*Specify independent variables, features extraction and dimension reduction, model, training and evaluation metrics.*
